# Patient Engagement Interventions to Improve Medication Management of Older Patients Across Transitions of Care: A Mixed Methods Systematic Review

**DOI:** 10.1111/jocn.70203

**Published:** 2026-01-26

**Authors:** Kelly Ottosen, Stephanie Garratt, Kerry Hwang, Grace Marconi, Pauline Wong, Gillian Kilby, Maneesh Prasad, Caitlin Deery, Elizabeth Manias

**Affiliations:** ^1^ Monash Nursing and Midwifery Monash University Clayton Australia; ^2^ Monash Health Clayton Australia

## Abstract

**Aims:**

Identify and describe patient engagement interventions used to improve medication management in older adults during transitions of care.

**Design:**

A mixed‐methods systematic review.

**Methods:**

A comprehensive search of all study designs was conducted. Studies were categorised using the ladder of patient and family engagement, a framework that positions engagement from low (passive) to high (active partnership) patient engagement.

**Data Sources:**

Six databases were searched from inception to April 2024.

**Results:**

The search yielded 29 reports, with 25 classified as studies. Most interventions (*n* = 19, 76%) were low‐level interventions that comprised informing patients in a passive manner. Interventions that facilitated high‐level engagement (*n* = 6, 24%) where patients were integrated in the decision‐making process were associated with consistently improved patient and healthcare long‐term outcomes.

**Conclusions:**

While low and high‐level engagement interventions were associated with significantly decreased hospital readmission rates, high‐level interventions consistently demonstrated positive patient outcomes. Interventions supporting older adults beyond discharge achieved meaningful and lasting patient and healthcare outcomes for older adults.

**Implications for the Profession and/or Patient Care:**

Findings provide clinical reference for designing engagement interventions, highlighting long‐term benefits of partnership‐based approaches and continuity beyond discharge.

**Impact:**

Engagement in medication management during transitions of care varied significantly. High‐level engagement was consistently linked to improved patient and healthcare outcomes but was often resource intensive. This review identifies the need to design balanced interventions that align with the preferences of older adults and real‐world contextual healthcare settings.

**Reporting Method:**

PRISMA guidelines.

**Patient or Public Contribution:**

No patient or public contribution.

**Protocol Registration:**

PROSPERO (registration number CRD42024557385).

## What Does This Paper Add to the Wider Global Clinical Community?

- Informing (low‐level) engagement interventions were linked to decreased hospital readmission rates.
- Collaborative (high‐level) engagement interventions were associated with sustained, long‐term patient and healthcare outcomes.
- Interventions that continue beyond discharge are required for long‐term and sustainable outcomes in older adults.

## 1 Introduction

For older adults admitted to the hospital as a result of illness or injury, this critical health event often encompasses transitions of care (Harvey et al. 2018). This journey may involve patient movements between different wards within the acute setting, transfers for procedural or diagnostic purposes, or movements to rehabilitation centres or long‐term aged care facilities (Manias et al. 2024). The term ‘transitions of care’ broadly describes a patient's transfer from one healthcare setting or level of care to another, which also includes interactions with multiple healthcare providers (Harvey et al. 2018). Transitions of care create challenges for ensuring appropriate and safe medication management and patient engagement. Older adults are frequently transferred between healthcare settings for specialised treatment, during which their medication regimens may be modified (Manias and Hughes 2015).

Older adults are particularly vulnerable to medication discrepancies during transitions of care due to their complex healthcare needs and polypharmacy (Naylor et al. 2013; Sawan et al. 2022). A medication discrepancy is defined as an unintended lack of consistency between two or more medication charts in relation to the documentation and information of each medication (Downes et al. 2015; Barnsteiner 2005). Medication discrepancies during transitions of care are associated with increased risk of preventable events that may lead to inappropriate medication use, patient harm or hospital readmissions (Bonnet‐Zamponi et al. 2013; World Health Organization 2019). The World Health Organization issued a technical report in 2019. This report highlighted the global nature of this issue and the critical safety risk medication discrepancies affecting patients worldwide during transitions of care (World Health Organization 2019). This report encouraged the need for interventions that actively engage patients as essential steps for mitigating medication discrepancies during transitions of care (World Health Organization 2019).

Interventions focused on patient engagement are associated with improved patient and healthcare outcomes, including increased patient satisfaction, medication adherence, self‐efficacy and decreased readmission rates (Marzban et al. 2022; Nilsen et al. 2022). However, effective methods of fostering active and meaningful engagement and the extent of older adult engagement, remain unclear (Daniel et al. 2020; Newman et al. 2021). Engagement encompasses healthcare professionals working in active partnership with patients in managing their medications across the healthcare system (Carman et al. 2013). Models such as the ladder of patient engagement can be used to classify engagement. The metaphor of a ladder is used to represent the continuum of patient engagement, with each ladder rung progressively building upon the previous one, indicating an increasing level of engagement (Kim et al. 2018). Levels of engagement on the ladder range from low level information exchange, through to full patient integration in decision‐making processes in higher levels of engagement (Kim et al. 2018; Newman et al. 2021). Current evidence suggests that patient engagement regularly occurs at low levels, through passive exchanges of information, rather than active involvement and integration in decision‐making processes (Daliri et al. 2019; Kim et al. 2018; Tobiano et al. 2019). Research shows that older adults are receptive to increased involvement in medication management across transitions of care (Manias et al. 2024; Ozavci et al. 2022), but evidence remains limited regarding the nature of engagement methods for older adults that translate into improved patient and healthcare outcomes (Newman et al. 2021; Tobiano et al. 2019).

Prior reviews highlight the significance of communicating medication information to older adults during transitions of care (Ozavci et al. 2021; Tobiano et al. 2019). Ozavci et al. (2021) identified a heavy focus on patient perspectives during the discharge and post‐discharge period, with little attention during the inpatient period. The use of continuity of care models has revealed post discharge follow‐up and the use of shared decision‐making are effective in reducing readmission rates in older adults' transition from hospital to home (Leithaus et al. 2022). However, Spencer and Punia (2021), identified a significant gap in current interventions available to improve patient communication during discharge and while in the community. Despite the known risks associated with medications during transitions of care, existing literature does not clearly demonstrate how current interventions impact older adults' engagement. Furthermore, there is much heterogeneity on the classifications of engagement (Ozavci et al. 2021). This review addresses these gaps by using a structured classification framework to evaluate the effectiveness of interventions.

## 2 Methods

### 2.1 Aims

The aim of this systematic review was to identify and describe patient engagement interventions used to improve medication management in older adults during transitions of care. The review was guided by the following research questions: (1) What patient engagement interventions are used to improve medication management during transitions of care? (2) How are these interventions delivered to engage older adults in the management of medications during transitions of care? and (3) What mechanisms facilitate or impede the effectiveness of patient engagement in medication management across transitions of care?

### 2.2 Design

The systematic review was conducted in accordance with Preferred Reporting Items for Systematic Reviews and Meta‐Analyses (PRISMA) statement (Page et al. 2021) Appendix S1 and methodological guidance for mixed methods reviews (Stern et al. 2020). The study protocol for this systematic review was registered with PROSPERO (registration number CRD42024557385).

### 2.3 Search Strategy

A specialist librarian with expertise in database searching assisted the development of a search strategy of six databases, including CINAHL, MEDLINE, PsycINFO, Embase, Emcare and Cochrane Central Register of Controlled Trials (CENTRAL). Articles published in English from inception to April 2024 were searched. Reference lists of included full‐text papers and forwards and backwards citation searches were also conducted to identify any further eligible studies. Four groups of key terms were searched individually and then combined using Boolean operators ‘OR’ and ‘AND’. These four groups of key terms were (i) patient engagement, (ii) interventions, (iii) medication management and (iv) transitions of care. Details of the search strategy and its outcomes are listed in Appendix S2.

Search results were imported into EndNote version 21 (Clarivate 2023) and then imported into Covidence (Covidence 2024), where duplicates were removed and screening and data extraction were conducted. Table 1 shows the inclusion and exclusion criteria for the systematic review. Two reviewers independently screened titles and abstracts against the inclusion exclusion criteria. Relevant reports underwent full‐text screening by two independent reviewers. Any discrepancies identified during the screening process were managed by consensus with the research team.

**TABLE 1:** Inclusion and exclusion criteria.

Inclusion	Exclusion
Participants were older adults aged ≥ 65 years	Studies primarily focused on families, caregivers, paediatric patients or healthcare professionals
Patients had transferred between at one acute care setting and another setting	Primary setting was palliative or hospice (end‐of‐life) care or the interventions commenced post‐discharge
Interventions initiated during the acute care admission, focussing on patient engagement in medication management throughout transitions of care	Publications that were not primary research or lacked full‐text access were excluded.
Outcomes examined patient or healthcare outcomes and could involve the use of any measurement method and time interval for these outcomes (e.g., hospital readmissions, emergency department visits, patient satisfaction, medication knowledge, understanding of condition and treatment and medication decision‐making)	Not published in English
Empirical research of any design	

### 2.4 Data Extraction, Synthesis and Quality Appraisal

Data extraction was conducted independently by two reviewers and consensus was performed by a third reviewer. Any discrepancies were addressed through discussion. A data extraction form was developed to align with the aims and research questions. This form aimed to capture key study information, including study design, population details, context, intervention, level of engagement, enabling factors, barriers and conclusions.

Data synthesis was conducted by one researcher using a three‐step convergent segregated approach, involving the independent analysis of quantitative and qualitative data, before integrating the results (Lizarondo et al. 2020; Stern et al. 2020). Inductive and deductive approaches were used in order to capture new insights and then illuminate findings by mapping against an established framework, the ladder of patient and family engagement (Kim et al. 2018).

Inductively, researchers first drew on the Thomas and Harden (2008) three‐step approach to guide the analysis. This approach focused on the identification and development of descriptive and analytical themes to offer new insights that move beyond summarising existing studies (Thomas and Harden 2008). All included papers were uploaded onto NVivo software (QSR International 2024). In step one, reviewers conducted a line‐by‐line reading and coding of relevant sentences to familiarise themselves with each paper's methods, population and key findings (Thomas and Harden 2008; Campbell et al. 2011). Step two involved the development of descriptive themes, based on the initial codes found in step one (Thomas and Harden 2008). Finally, analytical themes were generated and examined in step three, as a means of going beyond the descriptive level and developing new insights.

**FIGURE 1:**
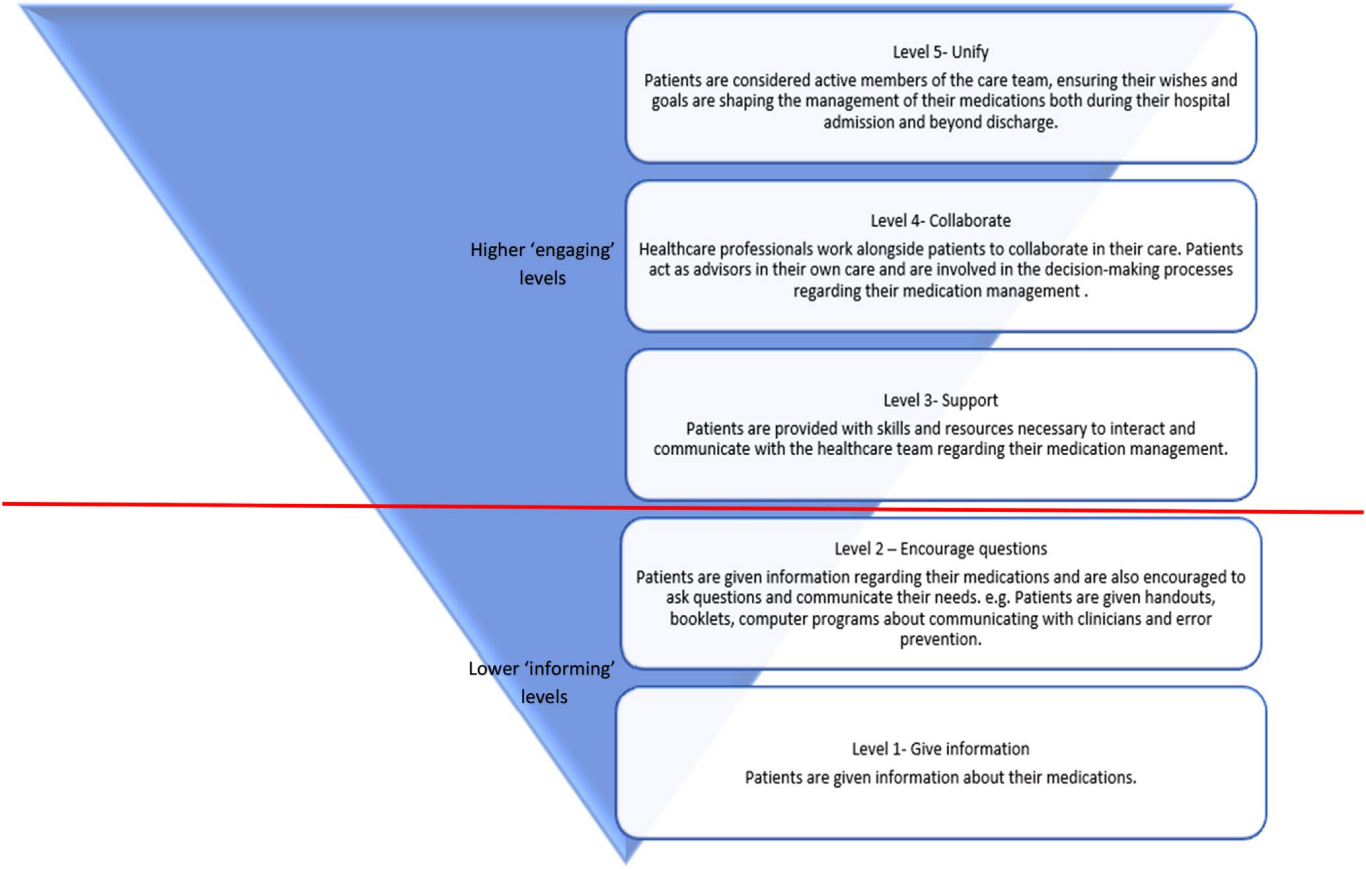
Levels of patient engagement. [Colour figure can be viewed at wileyonlinelibrary.com]

These inductive themes were then mapped deductively against the ladder of patient and family engagement, which provided a framework to situate the findings within established levels of engagement (Kim et al. 2018). Figure 1 illustrates an adapted version of the Kim et al. (2018) ladder. The ladder emphasises how each level builds and expands from the previous, fostering more comprehensive and integration forms of patient engagement. Levels one and two are considered low (informing) levels of engagement, describing information sharing or one‐way interactions with patients on a passive level. Patients may receive handouts or information material during this engagement and ask questions. Levels three, four and five are considered high (engaging) levels of engagement. These levels aim to empower patients with skills or tools necessary to contribute and collaborate in their care by partnering with the healthcare team. Level five is the highest level of engagement. At this level, patients are considered equal partners in decision‐making processes and actively partner with the healthcare team in co‐designing care plans and patient driven goals. To ensure rigour, generated codes and themes underwent regular review by the research team in developing and refining themes (Campbell et al. 2011).

Meta‐analysis was conducted using Cochrane Review Manager 8.1 on studies with similar outcomes (Cochrane 2020). Study heterogeneity was assessed using the Cochran's Q (Chi^2^) test and *I* ^2^ statistic. Tau^2^ was estimated using the DerSimonian and Laird (DL) method, applied to account for study variance. Combined dichotomous data were analysed using the odds ratio. Outcome effects were combined using a Mantel–Haenszel random‐effects model.

Quality appraisal and risk of bias were conducted independently by three researchers. Any discrepancies were resolved through consensus with the whole research team. The quality of all included studies was assessed using the Mixed‐Methods Appraisal Tool (MMAT) (Hong et al. 2018). Studies were included regardless of their MMAT score. Non‐randomised controlled studies were evaluated using the Risk of Bias in Non‐randomised Studies of Interventions (ROBINS‐I) tool (Sterne et al. 2016). The Cochrane Collaboration tool was used to assess risk of bias in randomised controlled studies (Higgins 2011). Results of quality appraisals and risk of bias are shown in Appendices S3 and S4.

## 3 Results

**FIGURE 2:**
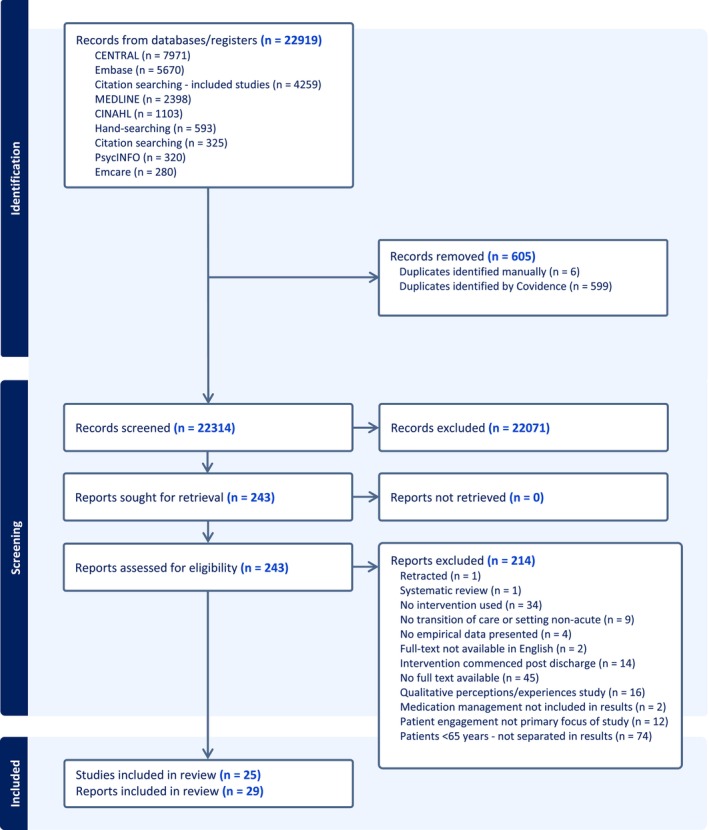
PRISMA flow diagram. [Colour figure can be viewed at wileyonlinelibrary.com]

In total, 22,919 records were imported for screening in Covidence, of which 605 duplicates were removed. Figure 2 depicts the PRISMA flow diagram. For the purposes of this review and in line with PRISMA 2020 terminology, reports refer to any article providing information about a particular study, regardless of methodology or format (Page et al. 2021). A study refers to the overarching research conducted, which may contain multiple reports within (Page et al. 2021). Twenty‐nine reports met full inclusion criteria and were included in the review. Twenty‐five of these were classified as studies. Johansen et al. (2022), Robinson et al. (2023) and Robinson et al. (2024) investigated the IMMENSE study. The interventions undertaken by Kempen et al. (2019) and Kempen et al. (2021) were part of the MedBridge intervention. Finally, the OPERAM intervention was reported by Thevelin et al. (2022) and Blum et al. (2021).

### 3.1 Study Characteristics

Studies were conducted in nine countries, primarily in the United States (13 studies, 52%), followed by three (12%) in Sweden, two (8%) in France and one (4%) study each in Belgium, Norway, South Korea, Spain, Switzerland and the United Kingdom. One study spanned multiple European countries (Blum et al. 2021). The most common design was randomised controlled trials (18 studies, 62%), followed by non‐randomised controlled quantitative studies (9 studies, 31%). One study was a mixed‐method approach (3.5%) and one was a qualitative design (3.5%). Study sample sizes ranged from 13 to 5729 participants.

Nineteen (66%) report outcomes focused on hospital readmission rates. Seven of these reports also included emergency department presentations. Four (14%) focused on patient medication knowledge and two (7%) reports focused on patient experience. One (3%) report outcome involved examination of medication adherence, the number of medication discrepancies post discharge, health‐related quality of life and cost effectiveness. Twenty‐two (88%) interventions were commenced during the patients' acute admission, whilst three (12%) were initiated on the day of discharge. Sixteen (64%) interventions extended beyond discharge where health professionals from either the hospital or community provided continued support for patients during the post‐discharge period. Appendix S5 presents a summary of the studies and their key characteristics.

### 3.2 Quality of Reports

The application of the MMAT resulted in 21 (72%) reports receiving a score of five out of five (Appendix S3). Of the 18 randomised controlled trial reports assessed for bias using the Cochrane Collaboration Bias tool, four were deemed to be high quality due to rigorous blinding and randomisation processes (Blum et al. 2021; Gillespie et al. 2009; Lee et al. 2023; Legrain et al. 2011). Nine non‐randomised reports were assessed using the ROBINS‐I tool. Six reports demonstrated low risk in participant selection, but unclear risk of bias in intervention classification (Anderson et al. 2005; Bajeux et al. 2022; Dedhia et al. 2009; Huckfeldt et al. 2019; Lazaro Cebas et al. 2022; White et al. 2013). Results of these quality appraisals are shown in Appendix S4.

### 3.3 Findings From Thematic Synthesis

**FIGURE 3:**
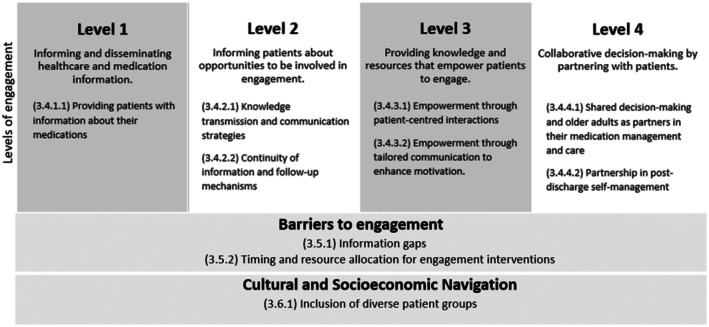
Coding framework (deductive) and inductive themes informed by the ladder of patient and family engagement (Kim et al. 2018) and Thomas and Harden's (2008) approach.

The ladder of patient and family engagement provided an established framework to classify four levels of engagement, which were mapped deductively to the inductive themes developed (Kim et al. 2018). No level five engaging interventions were identified, with the integration of older adults as full and active members of the healthcare team. As per Thomas and Harden's (2008) approach, inductive descriptive themes were identified and aligned with each level of the ladder of patient and family engagement. In addition, two inductive analytical themes were also found, highlighting their applicability to any type of intervention and contextual influences beyond the deductive framework. Figure 3 provides a visual representation of the themes and subthemes.

### 3.4 Level of Patient Engagement of Interventions

**FIGURE 4:**
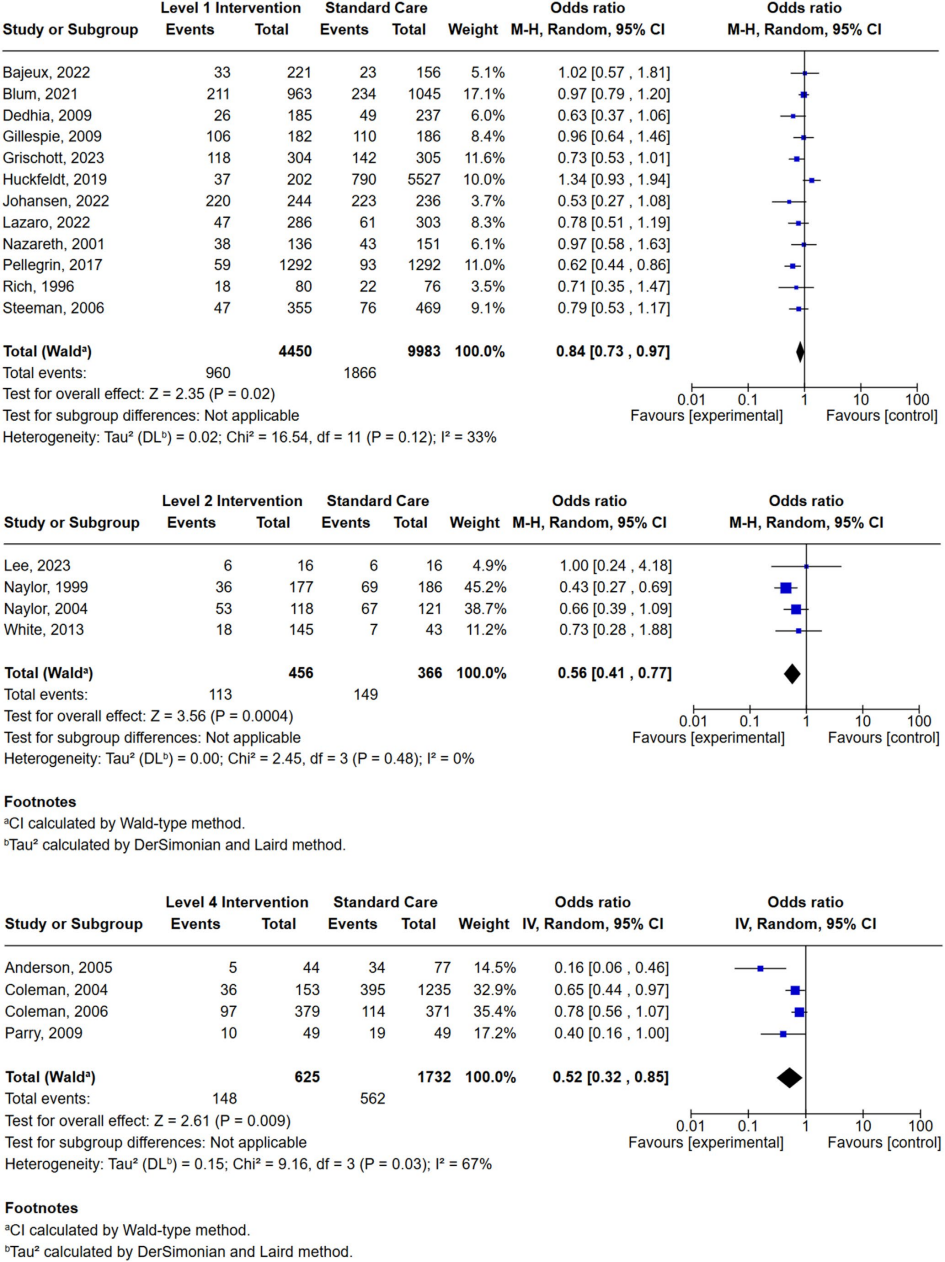
Meta‐analysis Forest Plot. [Colour figure can be viewed at wileyonlinelibrary.com]

Interventions were classified based on the ladder of patient and family engagement as shown in Figure 1 (Kim et al. 2018). According to the ladder, there were no interventions categorised as level five. Table 2 shows a summary of each report and the interventional components. Interventions were mostly multi‐modal in nature and involved the use of different types of strategies or approaches to engage older adults in medication management across transitions of care. There were 21 reports that comprised analysis of hospital readmission rates and were included in the meta‐analysis (Figure 4). There was no evidence of publication bias.

**TABLE 2:** Summary of multimodal intervention components and level of engagement.

Author, year, country	Sample size	Intervention components							Primary outcome	Statistical significance or finding	Secondary outcome	Statistical significance or finding
		Patient education	Medication reconciliation or review	Use of specialist nurse or healthcare professional	Health record, care plan or medication support tool/device	Shared decision making	Care transitions and discharge planning (Inc counselling)	Follow up home visit or telephone				
Level 1—Informing patients about medications												
Bajeux et al. (2022), France	377 156 (C) 221 (I)		✓				✓		Death, readmission rates or ED presentations 30 days post DC	NS (OR 1.6, 95% CI [0.7–3.6], *p* = 0.239) MRa 5.8% versus MRa + MRd 9%	Medication knowledge 30 days post DC Pt satisfaction with discharge process 7 days post DC	No difference between the groups MRa 67.6% versus MRa + MRd 80.8, *p* = 0.003
Blum et al. (2021), Europe (OPERAM)	2008 1045 (C) 963 (I)		✓			✓			Drug related hospital readmission rates 12 months post DC	NS (HR 0.95, 95% CI [0.77–1.17]) *p* = 0.62 Cont 22.4% versus inter 21.9%	QOL at 12 months post DC (EQ‐VAS) Medication adherence at 12 months post DC (MMAS‐8)	NS (OR 2.29, 95% CI [0.31–4.26]) *p* = 0.02 Mean (cont vs. inter): 19.1 versus 17.8 (OR 0.03, 95% CI [−0.05–0.12]) *p* = 0.46 Mean (cont vs. inter): 0.815 versus 0.759
Dedhia et al. (2009), United States	422 237 (C) 185 (I)						✓		Readmission rates 30 days post DC	↓ (OR 0.59, 95% CI [0.34–0.97]) Cont 21.9% versus inter 14.1%	ED presentations 30 days post DC	↓ (OR 0.58, 95% CI [0.34–0.98]) Cont 21.1% versus inter 13.5%
Gillespie et al. (2009), Sweden	400 201 (C) 199 (I)	✓						✓	Readmission rates 12 months post DC	NS (OR 0.96, 95% CI [0.64–1.46]) Quotient: cont 59.1 versus inter 58.2	Cost per patient for ED presentations and hospital readmissions	Cont $12,500 versus inter $12,100
Grischott et al. (2023), Switzerland	609 305 (C) 304 (I)		✓						Readmission rates 6 months post DC	NS (log rank test, *p* = 0.28) Cont 47% versus inter 39%	ED presentations 6 months post DC	NS (HR 1.14, 95% CI [0.63–2.09], *p* = 0.66)
Huckfeldt et al. (2019), United States	5729 5527 (C) 202 (I)						✓		Readmission rates or ED presentations 30 days post DC	NS (difference 3.5 pp., 95% CI [−2.0–8.9] *p* = 0.21) Cont 14.3% versus inter 18.3%	Readmission rates or ED presentations 7 days post DC	NS (difference 1.0 pp., 95% CI [−2.1–4.0] *p* = 0.54) Cont 4.9% versus inter 5.9%
Johansen et al. (2022), Norway (IMMENSE)	516 257 (C) 259 (I)						✓		Readmission rates 12 months post DC ED presentations 12 months post DC	NS (Incidence rate ratio 1.01, 95% CI [0.78–1.30]) Cont *n* = 223 versus inter *n* = 220 NS (Incidence rate ratio 1.02, 95% CI [0.78–1.33]) Cont *n* = 276 versus inter *n* = 277	All‐cause mortality 12 months post DC	NS (OR 1.06, 95% CI [0.67–1.69]) Cont 19.5% versus inter 19.7%
Lazaro Cebas et al. (2022), Spain	589 303 (C) 286 (I)		✓			✓	✓		Readmission rates 30 days post DC	NS [OR 0.76, 95% CI 0.495–1.166] (*p* = 0.209) Cont 20.13% versus inter 16.43%	Cost savings of the intervention per readmission	€1301.26 saving for all study pts
Nazareth et al. (2001), UK	362 181 (C) 181 (I)						✓	✓	Readmission rates 3 months post DC	NS (Proportional difference 18%, 95% CI [−10.6%–10.2%]) Cont 39.2% versus inter 39%	Knowledge of medications 3 months post DC No. of deaths 3 months post DC	NS (mean difference 0.07, 95% CI [−0.0032–0.173]) Mean: cont 0.62 versus inter 0.69 NS (Proportional difference 3.26%, 95% CI [−1.5%–7.7%]) Cont 2.8% versus inter 6.1%
Pellegrin et al. (2017), United States	1291		✓				✓	✓	Medication related hospitalisation rate per 1000 admissions	↓ (standard error 1.71, 95% CI [1.07–7.80], *p* = 0.01)	Annual cost‐savings of avoided admissions	$6.6 million
Rich et al. (1996), United States	156 76 (C) 80 (I)	✓						✓	Medication adherence 30 days post DC	↑ (*p* = 0.003) (does not state stat method) Cont 81% ± 17.2% versus inter 87.9% ± 12.0%	Readmission rates 3 months post DC	↓ (Fisher's exact test, *p* = 0.087) Cont 28.9% versus inter 22.5%
Robinson et al. (2023), Norway (IMMENSE patients' subset)	285 137 (C) 148 (I)						✓		Cost per patient	Cont €24,717, 95% CI [21,079‐28,957] versus inter €29.147, 95% CI [25,181‐33,654]	—	
Robinson et al. (2024), Norway (IMMENSE patients' subset)	285 137 (C) 148 (I)						✓		Health related quality of life (HRQoL) over 12 months from acute hospitalisation	NS (EQ‐5D‐3L) Index score: cont 0.482 versus inter 0.495	—	
Steeman et al. (2006), Belgium	824 469 (C) 355 (I)			✓	✓		✓		Readmission rates 3 months post DC	NS (Adjusted OR 0.47, 95% CI [0.31–0.70], *p* = 0.66) Cont 16.2% versus inter13.2%		
Thevelin et al. (2022), Europe (OPERAM patients' subset)	48 MM		✓			✓			Patient experience	NA	NA	NA
Level 2—Informing patients about ways to engage and communicate with clinicians												
Esposito (1995), United States	42 G1–11 (C) G2–8 G3–10 G4–14	✓						✓	Self‐administer medications and increased knowledge	↑ (no stat method stated)	Medication adherence scores	↑ (no stat method stated)
Kempen et al. (2019), Sweden (MedBridge patients' subset)	15 QUAL		✓					✓	Patient experience	NA	NA	NA
Kempen et al. (2021), Sweden	2637 892 (C) 922 (I1) 823 (I2)		✓				✗	✓	Readmission rates 12 months post DC	Cont versus inter (I1) NS (ARR 1.04, 95% CI [0.89–1.22]) Cont versus inter (I2) NS (ARR 1.15, 95% [0.98–1.34]) Rate ratios: cont 1.63, inter (I1)‐ 1.74, inter (I2)‐ 1.95	ED presentations 12 months post DC	Cont versus inter (I1) NS (ARR 1.16, 95% CI [0.94–1.44]) Cont versus inter (I2) NS (ARR 1.29, 95% CI [1.05–1.59]) Rate ratios: cont 0.71, inter (I1)‐0.84, inter (I2)‐0.97
Kennedy (1990), United States	65 33 (C) 32 (I)	✓							Medication knowledge 30 days post DC	(Pearson correlation coefficient, *r* = 0.8004, *p* < 0.0001)	Medication error rates 30 days post DC	(Pearson correlation coefficient, *r* = 0.‐315, *p* < 0.04)
Lee et al. (2023), South Korea	32 16 (C) 16 (I)	✓					✓	✓	Discharge readiness Knowledge preparation	NS (*t* = 0.346, *p* = 0.732) Mean: cont 136.56 versus inter 140.06 ↑ (t = 2.076, *p* = 0.046) Mean: cont 50.25 versus inter 58.13	Readmission rates 3 months post DC ED presentations 3 months post DC	NS (*t* = 0.00, *p* = 1.000) Cont 42.86% versus 42.86% inter NS (*t* = 5.057, *p* = 0.049) Cont 30.77% versus 0% inter
Naylor et al. (2004), United States	239 121 (C) 118 (I)	✓		✓		✓	✓	✓	Time to first readmission or death 12 months post DC	↓ (log rank χ^2^ = 5.0, *p* = 0.026)	Self‐reported QOL 3 months post DC Patient satisfaction 6‐weeks post DC	↑(*p* ≤ 0.05) Mean ± SD: cont 2.7+/− 1.5 versus inter 3.2 ± 1.5 ↑ (*p* ≤ 0.001) Mean ± SD: cont 77.8 ± 11.2 versus inter 83.1 ± 9.6
Naylor et al. (1999), United States	363 186 (C) 177 (I)	✓		✓		✓	✓	✓	Readmission rates 6 months post DC Time to first readmission	↓ (*p* ≤ 0.001) Cont 37.1% versus inter 20.3% ↑ (log rank χ^2^ = 11.1, *p* ≤ 0.001)	Cost savings 6 months post DC Depression Patient satisfaction	(*p* ≤ 0.001) Cont $1.2 million versus inter $0.6 million NS (*p* = 0.20) NS (*p* = 0.92)
White et al. (2013), United States	276	✓		✓				✓	Medication knowledge retention	↑ (*p* ≤ 0.001 min spent on teach back)	Readmission rates 30 days post DC	NS (*p* = 0.775)
Level 3—Empowering patients to engage with clinicians												
Al Musawi et al. (2024), Sweden	13		✓		✓	✓	✓	✓	Medication discrepancies	10/24 discrepancies corrected at discharge	Medication adherence Attitude	7/12 participants ↑ adherence scores (MARS‐5) 10/12 participants had lower concern scores (BMQ‐S)
Legrain et al. (2011), France	665 348 (C) 317 (I)	✓	✓						Readmission rates & ED presentations 3 months post DC	↓ (RRR 24.6%, 95% CI [2.2%–46.1%], *p* = 0.03) Cont 30.5% versus inter 23%	Readmission & ED presentation rates at 6 months	NS (RRR 13.5%, 95% CI [−1.9%–12.9%], *p* = 0.15) Cont 40.8% versus inter 35.3%
Level 4 —Partnering with patients in their care												
Anderson et al. (2005), United States	121 77 (C) 44 (I)	✓		✓		✓	✓	✓	Readmission rates at 6 months	↓ *p* = 0.01 (does not state stat method) Cont 44.2% versus inter 11.4%	Utilisation of home health care services Cost	50% more in control group Estimated cost savings $91,000 for 44 patients
Coleman et al. (2004), United States	1393 1235 (C) 158 (I)	✓	✓		✓	✓		✓	Readmission rates 30 days post DC Readmission rates 2 months post DC Readmission rates 3 months post DC	↓ (OR 0.52, 95% CI [0.28–0.96], *p* = 0.04) Cont 13.8% versus inter 8.9% ↓ (OR 0.43, 95% CI [0.25–0.72], *p* = 0.002) at 90 days Cont 22.9% versus inter 13.5% ↓ (OR 0.57, 95% CI [0.36–0.92], *p* = 0.02) Cont 32% versus inter 22.9%	Return to the ED within 30 days Level of confidence in medication regimes	NS (OR 0.76, 95% CI [0.44–1.30], *p* = 0.40) Cont 14.2% versus inter 11% ↑ Inter patients reported high levels of confidence
Coleman et al. (2006), United States	750 371 (C) 359 (I)	✓	✓		✓	✓		✓	Readmission rates 30 days post DC Readmission rates 3 months post DC Readmission rates 6 months post DC	↓ (OR 0.59, 95% CI [0.35–1.00], *p* = 0.048) Cont 11.9% versus inter 8.3% ↓ (OR 0.64, 95% CI [0.42–0.99], *p* = 0.04) Cont 22.5% versus inter 16.7% NS (OR 0.80, 95% CI [0.54–1.19], *p* = 0.28) Cont 30.7% versus inter 25.6%	Readmission for same condition 3 months post DC Cost saving 6 months post DC	↓ (OR 0.50, 95% CI [0.26–0.96], *p* = 0.04) Cont 9.8% versus inter 5.3% Cont $2546 versus inter $2058
Parry et al. (2009), United States	98 49 (C) 49 (I)	✓		✓	✓	✓		✓	Readmission rates 30 days post DC Readmission rates 3 months post DC	NS (*p* = 0.15) Cont 16.7% versus inter 6.8% ↓ (*p* = 0.01) Cont 31% versus inter 9.3%	Achieving self‐identified goals	Cont 30.8% versus Inter 37.5%

Abbreviations: ↑, increased; ↓, decreased; ✓, Component of intervention with patient involvement; ARR, adjusted risk ratio; Cont, control; DC, discharge; EQ‐VAS, European quality of life‐5 dimensions questionnaire; HR, hazard ratio; Inter, intervention; MMAS‐8, Measured using medication adherence questionnaire; NS, no change/not statistically significant; OR, odds ratio; pp, percentage points; QOL, Quality of life; RRR, relative risk ratio.

#### 3.4.1 Level 1—Informing and Disseminating Healthcare and Medication Information

Level one consisted of 12 reports aimed at informing or providing older adults with information about their medications. Older adults were often informed about their medications at various timepoints during their hospital admission, where consumer information leaflets were provided and at discharge when medication lists were provided. Meta‐analysis of 12 reports showed level one engagement interventions demonstrated a statistically significant reduction in hospital readmissions (OR 0.84, 95% CI [0.73–0.97]). Heterogeneity was moderate (*I* ^2^ = 33%) (Figure 4). Eight of the 12 reports were multi‐modal interventions and incorporated one or more components. In three reports, there were statistically significant results in primary outcomes relating to hospital readmission rates, medication‐related readmission rates, and medication adherence (Table 2).

Dedhia et al. (2009) found intervention patients had 41% lower odds of readmission when compared to the pre‐intervention group (OR 0.59, 95% CI [0.34–0.97]). Pellegrin et al. (2017) also demonstrated medication‐related hospital readmission was 36.5% lower in the intervention group (95% CI [1.07–7.80]) and medication‐related hospitalisation rates decreased by 4.4 more per 1000 admissions per quarter in intervention hospitals compared to non‐intervention hospitals (Pellegrin et al. 2017). Rich et al. (1996) reported increased medication adherence rates of 87.9% (SD ±12.0) in intervention patients, compared to 81.1% (SD ±17.2) in the control group (*p* = 0.003).

##### 3.4.1.1 Providing Patients With Information About Their Medications

Five reports comprised examination of the effects of medication reconciliation or review on patient engagement. Grischott et al. (2023) was the only report to incorporate medication reconciliation as a single intervention and found no significant reduction in hospital readmission or ED presentation at 1, 3 or 6 months post discharge. Medication reconciliation in their work was undertaken via patient interview to ensure the medication list was accurate and current (Grischott et al. 2023). Similarly, Bajeux et al. (2022) incorporated medication reconciliation via interview with the patient at both admission and discharge, whilst also including information sharing at the patient's discharge in the form of consultation. Steeman et al. (2006) utilised trained social workers or nurses to create patient care plans without patient involvement. The information contained within the care plan was provided to patients in order to promote continuity of self‐care. Bajeux et al. (2022) showed no statistical difference in combined unplanned readmissions and emergency department visitations between groups (OR 1.6, 95% CI [0.7–3.6]). Steeman et al. (2006) found intervention patients had 14.9% lower odds of requiring long‐term care facility placement compared to 23.7% in control group patients (adjusted OR 0.47, 95% CI [0.31–0.70]), after adjusting for baseline characteristic variations.

Employing a qualitative approach, experiences and perceptions were examined in patients participating in an intervention to increase engagement in medication management (Blum et al. 2021; Thevelin et al. 2022). The intervention featured the use of a multidisciplinary team approach where both doctors and pharmacists interviewed patients individually to identify medication discrepancies and make recommendations for improvement (Blum et al. 2021). A medication report that contained prescription recommendations and an ongoing medication plan was created and the patient was informed of any changes (Blum et al. 2021). Despite the reported discussions regarding medication changes performed with patients at discharge, patients still described a lack of information and communication regarding these medication changes. According to a patient:

No‐one explained anything to me! When I was discharged they just told me, so you've got this and that and this instead of that. And that's all. As for the whys and wherefores, I've no idea.

(Thevelin et al. 2022, 891)

Patient counselling and the use of follow‐up telephone calls or home visits were components of multi‐modal interventions found in two reports (Gillespie et al. 2009; Rich et al. 1996). Information provided to patients during their acute care stay involved verbal communication about the importance of medication adherence, understanding side effects and correct administration of medications (Gillespie et al. 2009), or by incorporating the use of a booklet used as a teaching guide (Rich et al. 1996). Gillespie et al. (2009) did not specify the number of education sessions provided, whilst Rich et al. (1996) stated patients were seen daily by the study nurse. Both reports comprised ongoing support post‐discharge where the research nurse or pharmacist telephoned the patient to ensure medication adherence. Rich et al. (1996) described achieving an overall 85% medication adherence rate in the intervention group, compared to 69.7% in the control group (*p* = 0.036), whilst Gillespie et al. (2009) found no statistical significance in the likelihood of hospital readmission rates between the groups (OR 0.96, 95% CI [0.64–1.46]).

Conversely, counselling occurred as a one‐off event at the time of the patient's discharge in three reports (Dedhia et al. 2009; Nazareth et al. 2001; Pellegrin et al. 2017). This education was undertaken in conjunction with a discharge planning meeting, where the pharmacist explained the medication plan (Nazareth et al. 2001; Pellegrin et al. 2017) or used written discharge instructions with large print font as an added education tool (Dedhia et al. 2009). Post‐discharge follow‐up was also included in these interventions, which included the community pharmacist making telephone calls to promote medication adherence (Pellegrin et al. 2017) or providing support in identifying medication discrepancies (Nazareth et al. 2001). Contact information of the discharge planning team was given to patients in one intervention (Dedhia et al. 2009). Pellegrin et al. (2017) reported an associated cost saving of USD $6.6 million in avoided readmissions due to the intervention. However, researchers noted the intervention to be time‐intensive as the discharge education and counselling were estimated to take up to 1 h per patient. Additionally, Nazareth et al. (2001) found no significant impact of their intervention on readmission rates and ED presentations 3 months post‐discharge. In addition, they did not find the intervention improved patients' medication knowledge post‐discharge.

#### 3.4.2 Level 2—Informing Patients About Opportunities to Be Involved in Engagement

Level two included seven reports that aimed to inform patients, whilst also encouraging them to communicate or ask questions. Meta‐analysis results of four reports showed level two engagement interventions demonstrated a statistically significant reduction in hospital readmissions (OR 0.56, 95% CI [0.41–0.77], *p* = 0.0004), with no significant heterogeneity across the included studies (*I* ^2^ = 0%) (Figure 4).

Five reports demonstrated statistically significant results in their primary outcomes. Naylor et al. (1999) found intervention patients were less likely to be readmitted to hospital at 6 months post‐discharge (20.3% vs. 37.1%, *p* ≤ 0.001) (Naylor et al. 1999). Similar findings are reported by Naylor et al. (2004), who observed that 47.5% of intervention patients had been readmitted to hospital 52 weeks post‐discharge, compared to the control group 61.2% (*p* = 0.01). Lee et al. (2023) found a meaningful improvement in medication knowledge between groups (*t* = 2.076, *p* = 0.046), whilst Kennedy (1990) reported a strong positive association between patient medication knowledge and medication administration skills (Pearson correlation coefficient, *r* = 0.8, *p* < 0.0001). Similarly, White et al. (2013) evaluated the impact of teach‐back on medication adherence using four standardised questions administered by nursing staff and reported that all patients who received education lasting 60 min or more answered all teach‐back questions correctly.

##### 3.4.2.1 Knowledge Transmission and Communication Strategies

The use of a specialist nurse or other healthcare professional to deliver the education throughout the patients' inpatient stay was featured in three reports (Naylor et al. 1999; Naylor et al. 2004; White et al. 2013). Education was used in conjunction with discharge planning and outpatient follow‐up, which involved designing and developing goals that encouraged patients to ask questions about their medications. Despite providing this opportunity to ask questions, post‐discharge interviews with patients examining communication and decision‐making processes during their admission revealed patients had problems with the delivery of the information provided (Kempen et al. 2019; Kempen et al. 2021). Patients expressed problems in receiving and retaining the information, with many feeling the information was not effectively communicated as they had ‘forgotten everything’ (Kempen et al. 2019, 154) shortly following the education sessions. Furthermore, a need to be more involved in the decision‐making process was highlighted, patients stating they were often left with many unanswered questions. According to a patient:

I sure did have questions, but […] I did not really know what the conversation was about.

(Kempen et al. 2019, 154)

Kennedy (1990) used education as a unimodal intervention. The number of education sessions provided was not specified by the researchers but were aimed at increasing patients' self‐care in medication by providing information on the prescribed medications. A comparison of four education methods was employed by Esposito (1995) in order to determine their feasibility. The control group received the standard education that included a medication and discharge summary sheet, group two received education and an additional 30 min of verbal instructions, group three received education and a medication schedule; and group four received education, an additional 30 min of verbal instructions and the medication schedule. It was found that the control group had a higher rate of medication errors (*n* = 5), compared to groups three (*n* = 1) and four (*n* = 1), who received education in conjunction with a medication schedule (Esposito 1995).

##### 3.4.2.2 Continuity of Information and Follow‐Up Mechanisms

The incorporation of follow‐up services, where a home visit or telephone call was conducted after patient discharge to answer any outstanding questions, was featured in two reports (Kempen et al. 2021; Lee et al. 2023). Patients could also telephone the care coordinators at any time of day for 12 weeks following discharge (Lee et al. 2023). Patients were able to ask further questions regarding their medications; they expressed receiving a lot of conflicting information from the hospital staff and their community healthcare professional. A patient commented:

It is quite interesting that one [family] doctor thinks you should take those [medications] and then you're here [at the hospital] and both a pharmacist and a doctor say ‘No, she will not have these medications, that is unnecessary’.

(Kempen et al. 2019, 154)

#### 3.4.3 Level 3—Providing Knowledge and Resources That Empower Patients to Engage

Level three consisted of two reports that included interventions aimed at empowering patients with skills and tools to communicate and engage in their own care. No meta‐analysis of level three engagement interventions was able to be performed, as the outcomes were dissimilar.

##### 3.4.3.1 Empowerment Through Patient‐Centred Interactions

Medication reconciliation and review featured as a multi‐modal interventional component across the two reports, where the patient was interviewed and encouraged to ask questions and discuss current and foreseeable problems with their medications (Al Musawi et al. 2024; Legrain et al. 2011). These interviews also included elements of shared decision‐making, where patients' short‐ and long‐term medication management goals were discussed, to construct a personalised plan that could be utilised post‐discharge from the acute setting (Al Musawi et al. 2024). Twenty‐four medication discrepancies were detected during the patients' acute admission; however, only 10 were corrected. Al Musawi et al. (2024) highlighted a primary reason for these 10 corrections was due to time constraints, leading to missed opportunities to accurately complete the medication discharge summary.

Two weeks following discharge, follow‐up interviews conducted by the pharmacist were performed in conjunction with monthly home visits or telephone calls, aimed at answering any questions and adjusting the patient's medication plan (Al Musawi et al. 2024). The pharmacist also offered a memory and dosing support dispensing medication device that was programmed to signal a light or sound at scheduled dosing times. Prior to the intervention, 12 of the 13 patients had negative attitudes as indicated by their Beliefs About Medicines Questionnaire Specific (BMQ‐S) scores (Al Musawi et al. 2024). Following the intervention, five of the 13 patients had negative scores (Al Musawi et al. 2024). Equally, prior to the intervention, eight patients had high adherence scores as per the Medication Adherence Report Scale (MARS‐5) scores. Following the intervention, this increased to 11 patients (Al Musawi et al. 2024).

##### 3.4.3.2 Empowerment Through Tailored Communication to Enhance Motivation

Tailored communication delivered via four inpatient sessions was offered in the report by Legrain et al. (2011). These sessions focusing on patients' preferences, values and treatment burden were designed to enhance the patients' motivation to engage in their medication management during admission and beyond discharge. Session one focused on linking the importance of medication adherence in relation to presenting any worsening health conditions. Sessions two and three involved providing patients with the skills and confidence necessary to identify any medication discrepancies they may find following discharge and also strategies on how to manage these situations. Lastly, session four was an optional session where patients could focus on specific medication topics (Legrain et al. 2011). Following the intervention, it was found 23% of intervention patients had at least one hospital readmission or emergency department presentation at 3 months post‐discharge, compared to the control group (30.5%) (RRR 24.6%, 95% CI [2.2%–46.1%]; ARR 7.5%, 95% CI [0.8%–14.2%], *p* = 0.03). Of note, Legrain et al. (2011) found no statistical significance in the same outcomes at 6 months post‐discharge (RRR 13.5%; AAR = 5.5%, 95% CI [−1.9% to 12.9%], *p* = 0.15).

#### 3.4.4 Level 4—Collaborative Decision‐Making by Partnering With Patients

Level four consisted of four reports that included patient engagement interventions aimed at healthcare professionals collaborating with patients to become actively involved in the decision‐making processes that would in turn impact their care. Meta‐analysis results showed all four reports demonstrated statistically significant results in their primary outcome. Level four engagement interventions demonstrated a significant reduction in hospital readmissions (OR 0.52, 95% CI [0.32–0.85], *p* = 0.009). Heterogeneity using the *I* ^2^ was 67%, indicating high variability across the studies (Figure 4).

As a result of these interventions, a decrease in hospital readmission rates from 44.2% to 11.4% at six months post‐discharge was described by Anderson et al. (2005) with no statistical method reported (*p* = 0.01). Coleman et al. (2004) and Coleman et al. (2006) found statistically significant decreases in the risk of readmission at 30 days post‐discharge (OR 0.52, 95% CI [0.28–0.96]; Coleman et al. 2004) and (OR 0.59, 95% CI [0.35–1.00]; Coleman et al. 2006). Similarly, Parry et al. (2009) noted a statistically significant decrease in readmission rates (*p* = 0.01), with 9.3% of intervention patients being readmitted 90 days post‐discharge, compared to 31% in the control group.

##### 3.4.4.1 Shared Decision‐Making and Older Adults as Partners in Their Medication Management and Care

The three reports which utilised healthcare records also incorporated shared decision‐making as a key feature (Coleman et al. 2004; Coleman et al. 2006; Parry et al. 2009). Using a nursing coach, healthcare records were introduced to patients as a comprehensive resource that enabled them to understand their medication, recognise discrepancies and advocate for their own healthcare needs where required (Coleman et al. 2004; Coleman et al. 2006; Parry et al. 2009). The healthcare records were patient‐centred, individualised documents or folders that contained essential information required for patients to collaborate and partner in their medication management. Documents such as medication lists, medication details, potential adverse reactions, care instructions and follow‐up appointment schedules were contained within these healthcare records. A section for patients to report and document questions or concerns was also found within the healthcare record (Coleman et al. 2004; Coleman et al. 2006; Parry et al. 2009). Additionally, education that included role plays on various situations that the patient may encounter post‐discharge was included (Coleman et al. 2004; Coleman et al. 2006).

Shared decision‐making was also incorporated through a specialist nurse who delivered one‐on‐one education to encourage shared decision making through a patient‐centred approach (Anderson et al. 2005; Parry et al. 2009). To complement the education sessions conducted at the bedside, an information pack was also given to each patient (Anderson et al. 2005). This pack included supplemental information on the patients' care, in addition to medication management such as follow‐up appointments and continued guidance on managing medications at home (Anderson et al. 2005). The combination of information packs and targeted education aimed to allow patients to become collaborators in the decision‐making processes throughout their admission and ensure continuity of care upon discharge. The goal was to enable patient empowerment and independence in their medication management, while also detecting any changes in their condition that may need adjustments in their prescribed medications during their acute stay and beyond discharge. The researchers observed a decrease in the use of home care services by the intervention group and overall hospital cost savings, with the control group requiring 50% more home care services (Anderson et al. 2005).

##### 3.4.4.2 Partnership in Post‐Discharge Self‐Management

Post‐discharge follow‐up, offered in the form of telephone calls or home visits, was provided in all four reports (Anderson et al. 2005; Coleman et al. 2004; Coleman et al. 2006; Parry et al. 2009). This follow‐up was intended for patients to clarify any concerns post‐discharge and to ensure the continued use of their healthcare record with community physicians (Anderson et al. 2005; Coleman et al. 2004; Coleman et al. 2006; Parry et al. 2009). Patients' self‐reported levels of confidence in their medication regimens increased because of the healthcare record (Coleman et al. 2004). Parry et al. (2009) found no significant differences in patients reporting achieving or exceeding their self‐reported medication management goals between the intervention and control groups.

### 3.5 Barriers to Engagement

Barriers to effective conduct of interventions centred around resource limitations and patient experiences during the interventions. These included ineffective communication, dismissive healthcare professionals' attitudes and time or workload allocation of the healthcare professionals carrying out the intervention. Negative patient experiences with healthcare professionals who failed to provide strategies that involved the patient in their care often discouraged active engagement and participation in the intervention (Thevelin et al. 2022; Kempen et al. 2019). Strategies such as insufficient time for questions to be answered, and the use of overly technical language during explanations of care plans or discharge instructions were commonly mentioned (Thevelin et al. 2022; Kempen et al. 2019). According to one patient:

I actually did not understand what she [the pharmacist] wanted.

(Kempen et al. 2019, 153)

#### 3.5.1 Information Gaps

Patients described ineffective communication as an overarching cause of their reluctance to engage and contributed to misinformation. Lack of understanding from ineffective communication often resulted in patients feeling more confused and anxious about managing their medications at discharge (Thevelin et al. 2022). Thevelin et al. (2022) highlighted the crucial relationship between effective communication, sufficient information and the importance of patients being informed about their medication plan upon discharge. One patient commented:

When you start asking why, sometimes I think they find it hard to explain things. They all have their drug lingo. And that's what's difficult to grasp at times.

(Thevelin et al. 2022, 891)

#### 3.5.2 Timing and Resource Allocation for Engagement Interventions

Resource limitations and the inability to deliver the intervention in accordance with the proposed protocol were described as a significant barrier to patient engagement and positive outcomes in many studies (Al Musawi et al. 2024; Gillespie et al. 2009; Johansen et al. 2022; Lee et al. 2023). Healthcare professionals described not having enough time and feeling rushed and therefore experiencing difficulties in providing a comprehensive discussion and missing opportunities to correct medication discrepancies at the time of discharge (Al Musawi et al. 2024). According to one patient:

We went home after lunch, but it was so messy and we were in a hurry.

(Kempen et al. 2019, 155)

Nazareth et al. (2001) estimated the average total time per patient of conducting the intervention was 5.5 h. Legrain et al. (2011) calculated the mean duration of the intervention to be 231.6 min, noting the considerable amount of time to be a limitation of the study. In research that involved incorporation of a specialist nurse or physician, investigators often reported positive healthcare outcomes but questioned the feasibility of having full‐time specialist staff conducting these interventions, due to resource variability and costs associated with employment across different healthcare systems (Anderson et al. 2005; Lee et al. 2023; White et al. 2013).

### 3.6 Cultural and Socioeconomic Navigation

Details about patients' demographic circumstances, cultural and socioeconomic backgrounds varied and were inconsistently reported across the literature. In some reports, there were detailed demographical insights of the patient sample (Al Musawi et al. 2024; Blum et al. 2021; Rich et al. 1996). In other studies, investigators addressed the need to recruit participants of diverse cultural and socioeconomic backgrounds in creating patient‐centred approaches as limitations (Blum et al. 2021; Thevelin et al. 2022; Esposito 1995). However, in most reports, there were unclear details of the demographic characteristics of the sample, or the demographic characteristics were simply described with little emphasis on how these individuals were influenced by the intervention.

#### 3.6.1 Inclusion of Diverse Patient Groups

Factors such as education levels, health literacy, language barriers, and social support systems were acknowledged as potentially impacting patient engagement (Al Musawi et al. 2024; Anderson et al. 2005; Thevelin et al. 2022). Yet, these were not explored in any depth, with some investigators excluding patient groups based on these factors. In 10 reports, patients were expected to speak the native language of the country to be included in the research and in six reports, patients were expected to reside within a certain geographical area to meet inclusion criteria. In one report, patients who resided outside a 10‐mile radius of the hospital were excluded from participation (Huckfeldt et al. 2019). This geographic restriction limited the broader population diversity and reduced variability in socioeconomic and demographic factors. It was also found that in four reports, patients were included if they were able to access telephone facilities upon discharge from the acute setting. Finally, authors of two reports identified their sample lacked diversity in race and social and health conditions (Anderson et al. 2005; Coleman et al. 2004).

## 4 Discussion

This review provides novel insights into how older adults are engaged in medication management during transitions of care. This is the first known systematic review to evaluate the level of engagement within interventions and the association with patient and healthcare outcomes. Many of these interventions were designed to inform or provide awareness to older adults about their medications through passive information exchanges or by encouraging questions (levels 1–2). Higher‐level interventions designed to engage older adults by empowering them with tools and skills to communicate and make informed decisions with the healthcare team (levels 3–4) were less common. Results revealed that irrespective of the level of engagement, both informing and engaging interventions were all associated with significant outcomes in reducing hospital readmission rates. No level five engaging interventions were identified, where older adults were integrated as full and active members of the healthcare team. There were clear barriers to intervention success and increased engagement with older adults across studies, such as time, cost of the intervention and lack of workforce availability to carry out the intervention. These barriers have repercussions for the development of future interventions that take up more complex approaches to engagement. Facilitating factors for success included continuity of care beyond discharge, and access to healthcare professionals through phone calls or home visits.

The breadth of interventions classified as informing (levels 1–2), across 19 out of 25 studies, reflects the widespread use of low‐level engagement with older adults (Kim et al. 2018). Interventions such as medication reconciliation and medication record counselling at discharge comprised the provision of medication handouts or information. However, these were delivered as a one‐way communication tool with older adults acting as passive recipients of information. The substantial use of informing interventions is consistent with prior work by Tobiano et al. (2019), who examined engagement in admission and discharge medication communication, finding communication was generally aimed at informing older adults. Irrespective of the level of engagement, significant decreases in hospital readmission rates were found in the meta‐analysis. These findings, however, should be interpreted with caution, as it was also found that informing level interventions often failed to extend into the community context, an issue linked as a key contributor to hospital readmissions (Spencer and Punia 2021). Additionally, patient outcome measures such as patient satisfaction or medication adherence were not measured consistently across studies and therefore did not feature in the meta‐analysis for this review. Consequently, these important aspects that could further provide insight into the effectiveness of these interventions remain unexplored and raise uncertainty around the extent of their benefits to patient outcomes.

Notably, none of the informing interventions reported patient outcomes related to shared decision‐making with older patients. Reliance on information exchange between older adults and healthcare professionals was shown in this review to, at times, have a negative effect on older adults' understanding of the management of their medications (Esposito 1995). This creates inconsistent results in patient outcomes such as quality of life, and medication knowledge (Bajeux et al. 2022; Robinson et al. 2024). Many older adults described a paternalistic role in decision‐making, or not being approached at all and having ‘no idea’ (Thevelin et al. 2022, 891). Taking steps to safeguard these patient outcomes is vital in ensuring the safety of older adults at post‐hospital admission (Mixon et al. 2014). Future research is needed to determine the impact of informing interventions on patient outcomes, in comparison to engaging interventions.

The limited number of higher‐level interventions that empowered and encouraged older adults to become collaborators in their care reflects missed opportunities for full integration of older adults into decision‐making processes. This is consistent with Kim et al.'s (2018) systematic review of medication safety interventions, where no level five interventions were found. Positioning older adults as recipients, rather than collaborators, may limit the extent to which the intervention aligns with their complex real‐world needs and preferences in managing their medications. Engaging older adults as collaborators in their own care through focused attention on their insights, preferences and lived experiences ensures care is truly patient‐centred (Jallow et al. 2023; Pereira et al. 2022). As shown in this review, considering these elements carefully is more likely to translate into positive and sustained healthcare and patient outcomes (Anderson et al. 2005; Coleman et al. 2006).

Furthermore, it is also necessary to critically examine whether the preference of older adults is to be fully integrated in decision‐making processes, as assumptions about their preferences may not coincide with empirical findings. Patient‐centred care models reflect full patient integration as the idealised approach (Kim et al. 2018), although this systematic review shows that the preferences of older adults may be highly variable and dependent on many contextual factors (Gluyas 2015; Kempen et al. 2019). In this review, factors such as patient fatigue, trust in healthcare professionals and the healthcare system and previous experiences within the healthcare system were all found to shape the preferences and willingness of older adults to engage (Kempen et al. 2019; Thevelin et al. 2022). These findings add to the current literature that describes the preferences of older adults as either proactive and wanting to be autonomous in their medication management (Bucknall et al. 2019), or wanting to take a passive role, trusting in the expertise of healthcare professionals (Naylor et al. 2013). Without a better understanding of these preferences and tailored methodologies to account for these varied preferences, unintended decision‐making responsibilities may be imposed onto older adults who do not wish for that responsibility. This undermines the concept of patient‐centred care.

Furthermore, there may be concerns around the feasibility of fully integrating older adults into decision‐making processes around medication management. This review revealed that engaging (high level) interventions typically relied on specialist healthcare professionals to deliver the protocol and many interventions were high‐cost and resource intensive (Anderson et al. 2005; Parry et al. 2009). Although not the focus of this review, other reviews have highlighted the important role that families can have in supporting older adults in medication management during transitions of care (Manias et al. 2019). If improved patient and healthcare outcomes can be achieved through less financially straining and workforce‐intensive methods that involve families, it raises questions about the necessity and practicality of these interventions. Without this evidence base, questions around the practicality and feasibility of such interventions remain.

Two key barriers for interventions aimed at engaging older adults in medication management were identified. Time constraints and workforce pressures were consistently identified as limitations and barriers for the conduct of interventions, in particular, those that included interventions which required use of healthcare professionals rather than independent research staff. Time pressures created a sense of urgency and feelings of being rushed, leading to miscommunication and misunderstanding for older adults and deviations from intervention protocols (Johansen et al. 2022; Nazareth et al. 2001; Al Musawi et al. 2024). Time is a valuable commodity; prior studies have found a significant relationship between the amount of time spent with older adults and their level of medication knowledge and willingness to engage around their medications (Carthon et al. 2019; McTier et al. 2015; White et al. 2013). Internationally, healthcare systems are under stress, particularly due to workforce shortages (Correia et al. 2025), which are a growing concern as both systemic and individual factors contribute to the current crisis. Healthcare professional burnout, increasing complexity of care, funding cuts and the aging patient population are considered the main elements putting strain on the healthcare system (Tamata and Mohammadnezhad 2023). As a result, time‐consuming or complex interventions, especially those that require action from healthcare professionals, may not be the most practical or feasible to enable better engagement and communication with older adults about their medication management at transitions of care (Jones and Dolsten 2024; Manias and Hughes 2015). Future research is needed around strategies to mitigate time pressures and increased workload demands to support the development of long‐term, sustainable strategies and interventions that promote engagement with older adults.

Continuity of care, where the intervention continued beyond discharge and into the community, was identified through this systematic review as an essential element to facilitate engagement of medication in older adults across transitions of care. Interventions incorporating scheduled telephone or home visit follow‐ups by healthcare professionals or access to healthcare professionals via telephone after older adults’ discharge demonstrated increased medication adherence and patient satisfaction (Anderson et al. 2005; Legrain et al. 2011). Transitioning from the hospital to home can be a chaotic experience as demonstrated by older adults facing substantial challenges in managing their medications (Kempen et al. 2019). Without post‐discharge guidance or corrective support, these factors can ultimately impact an older adult's recovery through the increased risk of poor medication adherence and subsequently increase the risk of hospital admissions or emergency department visits (Bauer et al. 2009; Pereira et al. 2022). Interventions that continued into the community led to older adults feeling more empowered to actively manage their medications, knowing a healthcare professional was accessible if they encountered any difficulties (Al Musawi et al. 2024; Esposito 1995; Naylor et al. 2004). These findings build on Pereira et al.'s (2022) work, reinforcing the importance of a community healthcare professional to follow up on older adults and the impact of integrating older adults into the management of their own medications. Additionally, these findings further emphasise Tomlinson et al.'s (2020) results, which suggested interventions that provided support to older adults for up to 90 days post‐discharge led to decreased hospital readmission rates. Continuity of care provides older adults with continued access to resources, ensures collaboration between healthcare professionals and older adults is maintained and allows for timely interventions for patients at high risk of readmission (Coleman et al. 2006; Takahashi et al. 2020). Considering these findings, future interventions should explore cost‐effective methods of providing continuity of care that align and address any recourse limitations within the healthcare system.

### 4.1 Limitations

This systematic review has limitations. Firstly, all reports were included in the review regardless of the quality or bias scores to ensure full insight into the phenomenon of interest was reported. Secondly, only papers written in English were included in the review. Lastly, although meta‐analysis was undertaken on hospital readmission, measurements were undertaken at varying time points (i.e., 3, 6 or 9 months post‐discharge) which may have impacted the consistency and comparability of the included data.

### 4.2 Impact for Research and Practice

The findings from this systematic review highlight that whilst communication was seen to be a core component of all interventions, many relied on informing older adults passively, rather than engaging them meaningfully. Future research should further explore the impact of informing (level 1–2) level interventions on patient specific outcomes and whether this translates into meaningful improvements in patient satisfaction and medication adherence. For practice, this highlights the need to employ interventions that are cost‐effective and can be integrated into routine care to create sufficient time for meaningful healthcare professional and older adult engagement, without increasing the workload for healthcare professionals.

With the absence of interventions that aim to fully integrate older adults in the management of their medications, a critical gap has been highlighted in current practice that reflects missed opportunities for true patient‐centred care and shared decision‐making. To facilitate the understanding of the impact of fully integrating older adults in decision‐making processes on healthcare and patient outcomes, future research should explore developing interventions that foster full collaboration.

Furthermore, interventions that extend beyond discharge have shown potential for positive outcomes. Continuing interventions such as post‐discharge phone calls, medication support, or home‐based check‐ins can identify any issues early and reduce the need for hospital readmissions, warranting the need for further research into sustained and cost‐effective strategies to support older adult engagement once they are in the community.

Lastly, many older adults come from diverse backgrounds and the incorporation of different population groups is vital for practical implications, as a one‐size‐fits‐all approach will fail to meet their specific needs. In practice, effective engagement interventions need to be adaptable and tailored to older adults' values, culture, levels of health literacy and social and economic background to improve engagement and promote equity in healthcare. Cognitive impairment and older adults living with dementia were not explicitly addressed in the included studies. Future research is needed around the feasibility of higher‐level interventions for these important population groups. Future research should be directed to include diverse population groups, particularly older adults from culturally and linguistically diverse backgrounds and those living with cognitive impairment.

## 5 Conclusions

The findings from this systematic review shed new light on how older adults are engaged in medication management during transitions of care. Many studies incorporated low level (informing) engagement interventions comprised of information exchange, resulting in positive healthcare outcomes but left questions on their impact on patient outcomes. High level (engaging) interventions revealed more consistent patient and healthcare outcomes but are limited by the time and workload constraints of healthcare professionals conducting the interventions. No level five interventions were identified in this review, raising questions on the need and practicality of such interventions in a healthcare system workforce that is already under stress and pressure. Future research requires balanced interventions that align with the preferences of older adults and the real‐world contextual healthcare setting.

## Author Contributions

Kelly Ottosen: Writing – original draft, Methodology, Investigation, Formal analysis, Data curation, Conceptualization. Stephanie Garratt: Writing – review and editing, Supervision, Project administration, Methodology, Data curation, Conceptualization. Kerry Hwang: Writing – review and editing, Methodology, Data curation, Formal analysis. Grace Marconi: Writing – review and editing, Methodology, Data curation, Formal analysis. Pauline Wong: Writing – review and editing, Methodology, Supervision. Gillian Kilby: Methodology, Data curation. Maneesh Prasad: Methodology, Writing – review and editing. Caitlin Deery: Methodology, Data curation. Elizabeth Manias: Writing – review and editing, Validation, Supervision, Funding acquisition, Data curation, Methodology, Data curation, Conceptualization.

## Funding

This work was supported by the National Health and Medical Research Council Investigator Grant (Project number: 2025864, Date of award: Jan 1 2024–31 Dec 2028). The sponsor had no role in the design, methods, data extraction, data synthesis, or preparation of the manuscript.

## Conflicts of Interest

The authors declare no conflicts of interest.

## Submission With Statistics

Kerry Hwang has completed a Master of Public Health, majoring in Epidemiology and Biostatistics. Elizabeth Manias has completed Master's level statistics subjects and has extensive experience in the conduct of meta‐analysis over several years.

## Supporting information


**Appendix S1:** Supporting Information.

**Appendix S2:** Supporting Information.

**Appendix S3:** Supporting Information.

**Appendix S4:** Supporting Information.

**Appendix S5:** Supporting Information.

## Data Availability

The data that support the findings of this study are available from the corresponding author upon reasonable request.
