# Supplementary material for: Patient Engagement Interventions to Improve Medication Management of Older Patients Across Transitions of Care: A Mixed Methods Systematic Review
Source: J Clin Nurs. 2026 Jan 26;35(6):2622–44. doi: 10.1111/jocn.70203 (PMC13156535; doi:10.1111/jocn.70203)
Supplement: Supplementary file 2 — Appendix S2: Supporting Information. [file JOCN-35-2622-s001.docx]

# Supplementary appendix 2: Sample search strategy (MEDLINE)

Ovid MEDLINE(R) ALL <1946 to April 09, 2024>

1. Patient Participation/ or Patient Advocacy/ or Decision Making, Shared/ or Patient Preference/ or Self Care/ or Self-Management/ or "Patient Acceptance of Health Care"/ or "Patient Education as Topic"/ or Empowerment/ or Personal Autonomy/ or Patient-Centered Care/ 266957
2. ((patient* or inpatient* or in-patient* or consumer*) adj5 (participat* or empower* or contribut* or engag* or centred or centered or activat* or collaborat* or advoca*)).mp. 251362
3. ((person* or user* or client*) adj3 (centred or centered or focused or driven or led)).mp. 21118
4. ((patient* or inpatient* or in-patient* or consumer*) adj2 (involv* or enabl* or partner* or consult* or input)).mp. 84611
5. ((patient* or inpatient* or in-patient* or consumer*) adj (focused or driven or led)).mp. 5975
6. (self-manag* or personali?ed care or patient-oriented).mp. 37642
7. ((patient* or consumer* or inpatient* or in-patient*) adj7 decision*).mp. 75282
8. ((share* or sharing) adj decision*).mp. 16698
9. ((patient* or inpatient* or in-patient* or consumer*) adj5 (willing* or intention* or preference*)).mp. 50782
10. ((patient* or inpatient* or in-patient* or consumer* or client*) adj3 (educat* or teach* or train* or learn*)).mp. 167482
11. 1 or 2 or 3 or 4 or 6 or 7 or 8 or 9 or 10 691568
12. Psychosocial Intervention/ or Internet-Based Intervention/ 2314
13. Quality Improvement/ or Total Quality Management/ or "Quality of Health Care"/ or Quality Assurance, Health Care/ 174108
14. Health Services Research/ or Comparative Effectiveness Research/ or exp Nursing Research/ or Outcome Assessment, Health Care/ or Pharmacy Research/ 174785
15. Health Care Evaluation Mechanisms/ or "Evaluation Studies as Topic"/ or "Validation Studies as Topic"/ or "Outcome and Process Assessment, Health Care"/ or Program Evaluation/ 218278
16. "Health Care Quality, Access, and Evaluation"/ or "Delivery of Health Care"/ or "Delivery of Health Care, Integrated"/ or Professional-Patient Relations/ or Nurse-Patient Relations/ or Physician-Patient Relations/ 272639
17. (quality adj5 (improv* or assurance*)).mp. 349092
18. (intervention* or program* or framework* or strateg* or initiative* or improv* or evaluat* or efficac* or effective* or feasib* or validation study).mp. 11807294
19. Qualitative Research/ or Focus Groups/ or "Interviews as Topic"/ or "Surveys and Questionnaires"/ or Health Care Surveys/ or Patient Reported Outcome Measures/ 749499
20. (qualitative* adj (research* or study or studies or design* or approach* or method*)).mp. 159291
21. ((patient* or inpatient* or in-patient* or consumer* or client*) adj5 (interview* or focus group* or survey* or questionnaire*)).mp. 157892
22. ((patient* or inpatient* or in-patient* or consumer* or client*) adj5 (perspective* or voice* or unvoiced or opinion* or view* or dialog* or perception* or perceiv* or experience*)).mp. 331223
23. ethnograph*.mp. 14689
24. Communication/ or Teach-Back Communication/ or communicat*.mp. 544127
25. 12 or 13 or 14 or 15 or 16 or 17 or 18 or 19 or 20 or 21 or 22 or 23 or 24 12722733
26. Medication Therapy Management/ or Self Medication/ or Self Administration/ or Prescription Drugs/ 26997
27. Medication Errors/ or Medication Reconciliation/ or Inappropriate Prescribing/ or "Drug-Related Side Effects and Adverse Reactions"/ or Polypharmacy/ or Potentially Inappropriate Medication List/ 62862
28. ((medicat* or medicine* or drug*) adj5 (safe* or error* or manag* or regime* or reconcil* or communicat* or continuity or discrepanc*)).mp. 143995
29. (adverse* and (reaction* or effect* or event*) and (medication* or drug* or medicine*)).mp. 1141646
30. ((inappropriate* or in-appropriate* or wrong* or error* or incorrect* or unnecessar*) adj5 (prescrib* or prescription* or medicat* or medicine* or drug* or polypharmacy or poly-pharmacy or dosing or dosage* or dispens*)).mp. 37236
31. 26 or 27 or 28 or 29 or 30 1273117
32. "Continuity of Patient Care"/ or Aftercare/ or "Hospital to Home Transition"/ or Patient Discharge/ or Patient Handoff/ or Patient Transfer/ or Transitional Care/ or "Transportation of Patients"/ 88251
33. ((transition* or transfer*) adj5 (care or home* or hospital* or ward* or unit* or department* or setting*)).mp. 49663
34. ((multiple or across) adj (setting* or site* or campus* or hospital* or ward* or unit* or department* or specialt* or discipline* or team*)).mp. 30244
35. ((continuit* or continuum*) adj5 care).mp. 35345
36. (hospital to home* or discharg*).mp. 366415
37. ((patient* or inpatient* or in-patient* or consumer* or client*) adj5 (handoff* or hand-off*)).mp. 2098
38. (handover* or hand-over* or aftercare* or after-care*).mp. 24950
39. 32 or 33 or 34 or 35 or 36 or 37 or 38 493704
40. 11 and 25 and 31 and 39 2561
41. limit 40 to english language 2464
