# Supplementary material for: Patient Engagement Interventions to Improve Medication Management of Older Patients Across Transitions of Care: A Mixed Methods Systematic Review
Source: J Clin Nurs. 2026 Jan 26;35(6):2622–44. doi: 10.1111/jocn.70203 (PMC13156535; doi:10.1111/jocn.70203)
Supplement: Supplementary file 3 — Appendix S3: Supporting Information. [file JOCN-35-2622-s003.docx]

# Supplementary appendix 3: Mixed Methods Appraisal Tool

| **RANDOMIZED CONTROL TRIALS** | | | | | | | | | | | | | | | | | | | |
| --- | --- | --- | --- | --- | --- | --- | --- | --- | --- | --- | --- | --- | --- | --- | --- | --- | --- | --- | --- |
|  | | Blum et al. 2021 | Coleman et al. 2004 | Coleman et al. 2006 | Esposito, 1995 | Gillespie et al. 2009 | Grischott et al. 2023 | Johansen et al. 2022 | Kempen et al. 2021 | Kennedy, 1990 | Lee et al. 2023 | Legrain et al. 2011 | Naylor et al. 2004 | Naylor et al. 1999 | Nazareth et al. 2001 | Parry et al. 2009 | Rich et al. 1996 | Robinson et al. 2023 | Robinson et al. 2024 |
| TOTAL SCORE | | 5 | 2 | 5 | 3 | 5 | 5 | 5 | 5 | 2 | 5 | 5 | 5 | 5 | 5 | 4 | 5 | 5 | 5 |
| **GENERAL**  **QUESTIONS** | Are there clear research questions? | Y | Y | Y | Y | Y | Y | Y | Y | Y | Y | Y | Y | Y | Y | Y | Y | Y | Y |
|  | Do the collected data allow to address the  research questions? | Y | Y | Y | Y | Y | Y | Y | Y | Y | Y | Y | Y | Y | Y | Y | Y | Y | Y |
| **RANDOMIZED CONTROLLED**  **TRIALS QUESTIONS** | Is randomization appropriately performed? | Y | U | Y | Y | Y | Y | Y | Y | Y | Y | Y | Y | Y | Y | U | Y | Y | Y |
|  | Are the groups comparable at baseline? | Y | N | Y | N | Y | Y | Y | Y | N | Y | Y | Y | Y | Y | Y | Y | Y | Y |
|  | Are there complete outcome data? | Y | Y | Y | Y | Y | Y | Y | Y | N | Y | Y | Y | Y | Y | Y | Y | Y | Y |
|  | Are outcome assessors blinded to the intervention provided? | Y | Y | Y | U | Y | Y | Y | Y | Y | Y | Y | Y | Y | Y | Y | Y | Y | Y |
|  | Did the participants adhere to the assigned intervention? | Y | U | Y | Y | Y | Y | Y | Y | N | Y | Y | Y | Y | Y | Y | Y | Y | Y |

| **NON-RANDOMIZED STUDIES** | | | | | | | | | | |
| --- | --- | --- | --- | --- | --- | --- | --- | --- | --- | --- |
|  | | Al Musawi et al. 2024 | Anderson et al. 2005 | Bajeux et al. 2022 | Dedhia et al. 2009 | Huckfeldt et al. 2019 | LazaroCebas et al. 2022 | Pellegrin et al. 2017 | Steeman et al. 2006 | White et al. 2013 |
| TOTAL SCORE | | 4 | 5 | 5 | 5 | 5 | 3 | 5 | 4 | 4 |
| **GENERAL**  **QUESTIONS** | Are there clear research questions? | Y | Y | Y | Y | Y | Y | Y | Y | Y |
|  | Do the collected data allow to address the research questions? | Y | Y | Y | Y | Y | Y | Y | Y | Y |
| **NON- RANDOMIZED CONTROLLED**  **TRIALS QUESTIONS** | Are the participants representative of the target population? | Y | Y | Y | Y | Y | Y | Y | Y | Y |
|  | Are measurements appropriate regarding both the outcome and intervention (or exposure)? | Y | Y | Y | Y | Y | Y | Y | Y | Y |
|  | Are there complete outcome data? | Y | Y | Y | Y | Y | N | Y | N | N |
|  | Are the confounders accounted for in the design and Analysis? | N | Y | Y | Y | Y | Y | Y | Y | Y |
|  | During the study period, is the intervention  administered (or exposure occurred) as intended? | Y | Y | Y | Y | Y | N | Y | Y | Y |

|  | | **MIXED-METHODS STUDIES** | **QUALITATIVE STUDIES** |
| --- | --- | --- | --- |
|  |  | Thevelin et.al. 2020 | Kempen et.al. 2020 |
| TOTAL SCORE | | 5 | 5 |
| **GENERAL**  **QUESTIONS** | Are there clear research  questions? | Y | Y |
|  | Do the collected data allow to address the  research questions? | Y | Y |
| **QUALITATIVE STUDIES** | Is the qualitative approach appropriate to answer the research question? |  | Y |
|  | Are the qualitative data collection methods adequate to address the research question? |  | Y |
|  | Are the findings adequately derived from the data? |  | Y |
|  | Is the interpretation of results sufficiently substantiated by data? |  | Y |
|  | Is there coherence between qualitative data sources, collection, analysis and interpretation? |  | Y |
| **MIXED METHODS STUDIES** | Is there an adequate rationale for using a mixed method design to address the research question? | Y |  |
|  | Are the different components of the study effectively integrated to answer the research question? | Y |  |
|  | Are the outputs of the integration of qualitative and quantitative components adequately interpreted? | Y |  |
|  | Are divergences and inconsistencies between quantitative and qualitative results adequately addressed? | Y |  |
|  | Do the different components of the study adhere to the quality criteria of each tradition of the methods involved? | Y |  |
