# Supplementary material for: Patient Engagement Interventions to Improve Medication Management of Older Patients Across Transitions of Care: A Mixed Methods Systematic Review
Source: J Clin Nurs. 2026 Jan 26;35(6):2622–44. doi: 10.1111/jocn.70203 (PMC13156535; doi:10.1111/jocn.70203)
Supplement: Supplementary file 4 — Appendix S4: Supporting Information. [file JOCN-35-2622-s004.docx]

# Supplementary appendix 4: Risk of Bias Appraisal Tool

| **RANDOMIZED CONTROL TRIALS**  **Cochrane** | | | | | | | | | | | | | | | | | | | |
| --- | --- | --- | --- | --- | --- | --- | --- | --- | --- | --- | --- | --- | --- | --- | --- | --- | --- | --- | --- |
|  | | Blum et al. 2021 | Coleman et al. 2004 | Coleman et al. 2006 | Esposito, 1995 | Gillespie et al. 2009 | Grischott et al. 2023 | Johansen et al. 2022 | Kempen et al. 2021 | Kennedy, 1990 | Lee et al. 2023 | Legrain et al. 2011 | Naylor et al. 2004 | Naylor et al. 1999 | Nazareth et al. 2001 | Parry et al. 2009 | Rich et al. 1996 | Robinson et al. 2023 | Robinson et al. 2024 |
| **Selection Bias** | Random sequence generation | L | U | L | L | L | L | L | H | L | L | L | L | U | L | L | L | L | L |
|  | Allocation concealment | L | U | U | M | L | U | U | L | L | L | L | L | L | U | U | U | L | L |
| **Performance Bias** | Blinding of participants and personnel | L | H | U | U | L | U | H | H | L | L | L | L | L | U | L | L | H | H |
| **Detection Bias** | Blinding of outcome assessment | L | L | L | U | L | U | L | L | L | L | L | H | L | L | L | L | L | L |
| **Attrition Bias** | Incomplete outcome data | L | L | L | H | U | L | L | L | H | L | L | L | L | H | L | L | L | L |
| **Reporting bias** | Selective reporting | L | L | L | H | L | L | L | L | L | L | L | L | L | L | L | L | L | L |

**L=Low Risk of Bias, H=High Risk of Bias, U=Unclear Risk of Bias*

| **NON-RANDOMIZED STUDIES**  **ROBINS-I** | | | | | | | | | | |
| --- | --- | --- | --- | --- | --- | --- | --- | --- | --- | --- |
|  | | AlMusawi et al. 2024 | Anderson et al. 2005 | Bajeux et al. 2022 | Dedhia et al. 2009 | Huckfeldt et al. 2019 | Lazaro Cebas et al. 2022 | Pellegrin et al. 2017 | Steeman et al. 2006 | White et al. 2013 |
| **Pre-intervention** | Bias due to confounding | H | H | H | U | U | U | M | M | U |
|  | Bias in selection of participants into the study | M | L | L | L | L | L | M | M | L |
| **At intervention** | Bias in classification of  interventions | L | U | U | U | U | U | U | L | U |
| **Post-intervention** | Bias due to deviations from intended interventions | L | L | M | L | H | M | L | L | L |
|  | Bias due to missing data | L | L | M | L | H | M | L | M | H |
|  | Bias in measurement of outcomes | M | M | L | M | M | L | L | M | L |
|  | Bias in selection of the reported result | L | L | L | L | L | L | L | L | L |

**L=Low Risk of Bias, H=High Risk of Bias, U=Unclear Risk of Bias*
