# Supplementary material for: Patient Engagement Interventions to Improve Medication Management of Older Patients Across Transitions of Care: A Mixed Methods Systematic Review
Source: J Clin Nurs. 2026 Jan 26;35(6):2622–44. doi: 10.1111/jocn.70203 (PMC13156535; doi:10.1111/jocn.70203)
Supplement: Supplementary file 5 — Appendix S5: Supporting Information. [file JOCN-35-2622-s002.docx]

# Supplementary appendix 5: Summary of reports and their key characteristics

| Author, Year | Country | Target Population | Mean Age | Transfer of Care | Intervention Details | Enablers | Barriers | Main Findings |
| --- | --- | --- | --- | --- | --- | --- | --- | --- |
| **Randomised Controlled Trials** | | | | | | | | |
| Blum et al. 2021  OPERAM | Switzerland, Netherlands Belgium, and Republic of Ireland | Multi-morbidity and poly-pharmacy | 79 | Acute Setting - Discharge Home | 1. Medication reconciliation - history taking with patient within 24-72 hours post admission.  2. STRIPA analysis - prescribing reformations (no with patient) 3. SDM (STOP/START criteria)- discussion with patient changes & plan created | - Not reported | - Complex medication regimes  - Suboptimal implementation of recommendations | 86.1% (n=789) intervention patients had inappropriate prescribing with a mean of 2.75 (SD 2.24) STOPP/START recommendations per patient.  At two months, 62.2% (n=491) had ≥1 recommendation implemented, primarily discontinuing inappropriate drugs  Drug-related hospital admissions were similar between groups and were not significantly reduced |
| Coleman et al. 2004 | United States | Conditions chosen due to likelihood of requiring post DC help | 75 (I)  78 (C) | From Acute Setting - Discharge Home | 1. Medication reconciliation  2. SDM - Personal Health Record to 'coach' and for self-advocation, identify discrepancies.  3. Patient education - role play  4. Follow up phone call- answer any further question post DC | - Simplicity and low cost of intervention | - Single site | Intervention patients half as likely (in odds) to readmit to hospital compared to control group  Return to ED- no statistical significance  Self-reported levels of confidence and understanding their medication regimen higher in intervention group |
| Coleman et al. 2006 | United States | Conditions chosen due to likelihood of requiring post DC help | 76 | From Acute Setting - Discharge Home or to skilled nursing facility | 1. Medication reconciliation  2. SDM - Personal Health Record to 'coach' and for self-advocation, also identify discrepancies.  3. Patient education - role play 4. Follow up phone call- answer any further question post DC | - Transition coach assumed a supportive role and was not a health care provider per se so was able to support more patients in this project | - High noncompliance rates Socioeconomic status  - Lower resource limitations | Intervention patients had lower hospital readmission rates than control subjects at each time interval.  Intervention patients were significantly less likely to be re-hospitalized at 90 days and 180 days for the same condition that precipitated the index hospitalization.  Hospital cost savings at 180 days - INT $2058, CONT- $2546 |
| Esposito, 1995 | United States | NA | 79 (I2)  75 (I3)  76 (I4)  74 (C) | From Acute Setting - Discharge Home; Discharge to Subacute care | 1. Education - 4 groups assessed using 4 different methods of delivery  2. Follow up visit for patient | - Clear instructions improve patients understanding  - Use of follow up to reinforce education | - “Too much confusion on the day of discharge"  - High level non-compliance - Small sample size (<25% of planned size) | Patients in groups I3 and I4 had less medication errors than in groups C and I2.  Adherence scores in groups C and I2 exhibited increased changes in the follow-up visits when compared with groups I3 and I4. |
| Gillespie et al. 2009 | Sweden | NA | 87 | From Acute Setting - Discharge Home | 1. Medication reconciliation- no patient involvement  2. Education throughout admission, with discharge counselling provided  3. Follow up visit for patient - follow up by nurse 2 months post DC | - Healthcare Professional Involvement | - Limited patient involvement  - Lack of Resources- 3 pharmacists needed  - Limited information about the extent of visits to primary care facilities during the follow-up | 16% reduction in all visits to the hospital and a 47% reduction in visits to the emergency department  Drug-related readmissions were reduced by 80%. Direct costs per patient for ED visits and readmissions: Total cost: Intervention: $12100, Control $12500 |
| Grischott et al. 2023 | Switzerland | NA | 77 | From Acute Setting - Discharge Home | 1. Medication reconciliation **–** some patient engagement  2. Education directed at pharmacy and doctors | - Not reported | - Losses to follow-up  - Incomplete medication data  - Self-declared outcomes  - Subjective measurement tools | At admission, 609 patients took a mean of 9.6 (4.2) drugs per patient.  Time-to-first-readmission no statistical significance  No clinically relevant differences between study arms at 1, 3, and 6 months after discharge. |
| Johansen et al. 2022  IMMENSE STUDY | Norway | NA | 83 | From Acute Setting - Discharge Home; community health setting | 1. Medication reconciliation – no patient involvement  2. Medication review - discussed with team (with patient if possible) to identify medication-related problems 3. Med List for DC Summary- no patient involvement 4. Patient Counselling (at DC) - medication list discussed Patient able to ask questions 5. Follow up - phone call to the patient’s general practitioner or nursing home physician/nurse (no patient involvement) | - Not reported | - High workload  - Lack of Resources  - Intervention complexity  - No follow-up with patient post DC | IMMENSE intervention did not have an effect on the rate of readmission or emergency department visits after 12 months |
| Kempen et al. 2021  MedBridge | Sweden | NA | 81 | From Acute Setting - Discharge Home; Discharge to Nursing Home; Discharge to Subacute care | 1. Medication Review at admission and DC  2. Education- practical use of medications, adverse effects, interactions, and the patient’s understanding 3. Follow up – (Intervention 2 only) phone call 2-7 days and 1-2 months post DC to check in (answer questions- provide counselling) | - Not reported | - Not reported | Unplanned hospital visits and readmissions did not differ between the two groups log-linear model.  Intervention group 2 who received follow-up was associated with an increased incidence of emergency department visits within 12 months.  There were no differences between treatment groups regarding other secondary outcomes |
| Kennedy, 1990 | United States | NA | 87 | From Acute Setting - Discharge Home; Nursing Home | 1. Education - self-care with aim to increase knowledge of prescribed medications and improve adherence (no of sessions not specified)  2. Follow up - observe retention and application of education (no patient involvement) | - Hands on education and mediation handling | - Limited diversity of population (mostly females) | A positive correlation noted between knowledge of medications and medication administration skills for all patients.  Patients who demonstrated high levels of medication administration skills sustained fewer medication errors  No significant relationship was found between patients’ knowledge of medication and percentage of medication errors committed. |
| Lee et al. 2023 | South Korea | Frail older patients with multiple health problems | 80 (I)  82 (C) | From Acute Setting - Discharge Home | 1. Education- provided at discharge medication review and chronic illness management 2. Follow up- home visit and six follow-up phone calls. Patients given phone number of transitional care coordinator they could call anytime 12/52 post DC | - Multidisciplinary team approach  - Individualised patient education  - Follow up visits to monitor for early detection of issues | - Resource intensive  - Insufficient staff and funding, limited ability to delivery program to full potential | No statistically significant difference in discharge readiness  Statistically significant in knowledge preparation  Community resource utilisation statistically significant at 12 weeks  All-cause readmission rate at 12 weeks was the same (42.86%) between the groups  Unplanned readmission rate 7.14 % in the experimental group and 35.71 % in the control group |
| Legrain et al. 2011 | France | NA | 86 | From Acute Setting - Discharge Home | 1. Medication review - interview and questions with patient  2. Education – delivered via 4 sessions with summary provided and self-follow up criteria constructed with the patient  3. Follow up- GPs contacted after the participant’s admission (no patient involvement) | - Multidisciplinary team approach  - Large multicentre - DC plan shared with GP to ensure continuity of care | - Resource/staff intensive | Statistically significant hospital readmission or ED visit rates at 3 months post DC  No significance at 6 months,  Event-free survival- No significance at 6 months |
| Naylor et al. 2004 | United States | Heart failure | 76 | From Acute Setting - Discharge Home | 1. Education – delivered by Advanced practice nurse within 24 hours of admission, then daily until DC. Develop goals and design DC plan 2. Follow up- phone calls or home visits post DC (at least 8 times). APN was able to be contacted anytime for 3 months post DC | - Utilisation of  - Advanced practice nurse to provide comprehensive personalised care and education.  - Regular follow ups.  - Goal setting | - Resource intensive with frequent visits and phone call availability  - Utilisation of advanced nurse, potential for limited transferability | Statistically significant time to first readmission or death longer at 52 weeks.  Patients had fewer readmissions and lower mean total costs ($7,636 vs $12,481).  Short-term improvements shown in overall quality of life at 12 weeks and patient satisfaction at 6 weeks |
| Naylor et al. 1999 | United States | Cardiac condition, respiratory infections, gastrointestinal procedures and lower extremity orthopaedic surgeries | 75 | From Acute Setting - Discharge Home | 1. Education- delivered by Advanced practice nurse within 48 hours of admission then daily visits. Individualised protocol developed with patient. Written instructions and medication schedule education provided  2. Follow up- 2 home visits within 48 hours post DC and 7-10 days post DC. APN was able to be contacted anytime for 3 months post DC - address questions or concerns, monitor patient progress and collaborate with GPs where needed  3. APNs made DC summaries at the end of intervention - sent to patients and GPs, detailing plans, goal progression and any ongoing concerns (no patient involvement) | - Utilisation of Advanced practice nurse to provide comprehensive personalised care and education.  - Frequent follow up visits and contactable healthcare staff at any time | - Resource intensive with frequent visits and phone call availability  - Utilisation of advanced nurse, potential for limited transferability | Statistically significant readmission rates at week 24, and fewer hospital days per patient.  Time to first readmission was increased in the intervention group.  Increased costs per patient associated with control $6661 vs $3630  No significance in post DC acute care visits, functional status, depression or patient satisfaction.  Mean patient satisfaction scores showed little change overtime; both groups remained highly satisfied with care |
| Nazareth et al. 2001 | UK | NA | 84 | From Acute Setting - Discharge Home | 1. Medication reconciliation (no patient involvement)  2. Counselling sessions at DC to explain DC plan and medications (purpose and appropriate doses of medication, liaising with GPs 2. Follow up- Visit by community pharmacist 7-14 days post DC. Check for discrepancies, assess understanding and adherence - 32-38mins for each visit | - Not reported | - High workload and time consuming (mean 5.5 hours to prepare and administer intervention per patient). - High patient drop out (50 from each group by 6-month follow up) | No significance in patients readmitted to hospital at 3- and 6-months.  No significant difference in deaths or outpatient or general practitioner attendance over 3 or 6 months.  Increased patient knowledge about their medication |
| Parry et al. 2009 | United States | NA | 81 (I) 83 (C) | From Acute Setting - Discharge Home, skilled nursing facility | 1. Personal Health Record  2. Education delivered by nurse transition coach to encourage PHR, self- manage medications, self-advocate, communicate their needs to staff and identify medication discrepancies  3. Follow up- Visit and phone calls with transition coach 48-72hrs post DC, 3 times up until 28 days post DC. Answer any remaining questions and remind patient to share and use PHR | - Healthcare professional involvement across all settings- established rapport and ongoing trust | - High intervention cost  - Single setting - High refusal rate from participates  - Socio-economic status lower | Intervention patients were less likely to be readmitted at 30, 90 and 180 days.  Patients reported achieving or exceeding their self-identified goal patients |
| Rich et al. 1996 | United States | NA | 81 (I) 78 (C) | From Acute Setting - Discharge Home | 1. Education- 15-page teaching book/guide. Patients visited daily by research nurse 2. Medication review (no patient involvement)  3. Follow up- phone calls by hospital homecare department and study nurse | - Multidisciplinary Team | - High non-compliance rates  - Socio-economic status lower - Resource limitations | Significantly better medication compliance rates Interventions patients had better adherence to medication regimens, fewer rehospitalizations and improved quality of life. |
| Robinson et al. 2023  IMMENSE | Norway | NA | 82 | From Acute Setting - Discharge Home | 1. Medication reconciliation - discussed with team and patient (if possible) 2. Patient Counselling- pharmacist gave patient medication list and discussed changes, reasons for changes. Patient able to ask questions 3. Follow up- phone call to the patient’s general practitioner or nursing home physician/nurse (no patient involvement) | - Not reported | - Not reported | Mean increased quality-adjusted life years 0.023. Increased additional healthcare costs per patient €4,429  Demonstrated cost-effectiveness primarily among patients (€824 per patient) with shorter hospital stays (< 14 days) |
| Robinson et al. 2024  IMMENSE | Norway | NA | 82 | From Acute Setting - Discharge Home; Nursing Home | 1. Medication reconciliation - discussed with team and patient (if possible) 2. Patient Counselling- pharmacist gave patient medication list and discussed changes, reasons for changes. Patient able to ask questions 3. Follow up- phone call to the patient’s general practitioner or nursing home physician/nurse (no patient involvement) | - Not reported | - Health-related quality of life (HRQoL) reference measurements were done at discharge, after intervention was initiated  - Limited sample size | EQ-5D-3L index scores significantly declined after 12 months  Short hospital stays had significant improvement 1-month post DC  The number of medications and receiving home-care services were the main factors associated with reduced HRQoL.  Intervention was not cost effective |
| **Non-randomised Controlled Trials** | | | | | | | | |
| Al Musawi et al. 2024 | Sweden | NA | 75 | From Acute Setting - Discharge Home; to Nursing Home; to Subacute care, community health setting | 1. Medication Reconciliation- reviewed with patient  2. SDM- personalised plan made with patient. Discussion of patient’s ‘narrative' about their medications. Short- and long-term medication goals  2. Follow up- Counselling and follow up interview 2 weeks post DC, participants offered one consult each month (home visit) or via phone, follow up on health plan and adjustments according to patient's needs Medication dispensing support given at patient’s preference | - Bilingual healthcare workers helped with overcoming barriers | - Lack of Resources- timely and costly follow up support  - Patients DC with medication discrepancies - physicians did not have time/missed the chance to correct the DC summary  - Language barriers  - Recruitment difficulties - Small population - Lack of control group - Self-reported questionnaires | Twenty-four medical discrepancies were found. Discharging physicians agreed that all discrepancies were errors- only ten were corrected in the discharge information.  Ten participants had a decreased total BMQ-S concern score (decreased anxiety) after the intervention  7 participants increased their total MARS-5 scores after the intervention (improved adherence). |
| Anderson et al. 2005 | United States | Patients diagnosed with congenital heart failure | 81 (I) 77 (C) | From Acute Setting - Discharge Home | 1. Education- delivered by specialist Heart Failure nurse at the bedside on all aspects of heart failure. Written information pack provided. Second session prior to DC 2. DC Planning- actionable plan individualised and discussed at DC. Included follow-up appointments and guidance on managing HF and meds at home 3. Follow UP- Home care nurses 6 week home clinical pathway (6-20) home visits and phone calls (15 mins) to reinforce education, address concerns and monitor adherence- provide guidance so patients continue to manage their medications, contribute to their own care and detect changes in their condition | - Multidisciplinary Team; Patient Empowerment (e.g. self-med admin) | - Lack of Resources- need specialist trained nurse may not be feasible everywhere  Small sample size - Lack of diversity - Decreased length of stay and acute nature of the illness (Congestive heart failure) | Statistically significant decrease in 30 day and 6-month readmission rate  Home health care visits significantly lower  Total cost saving for all 44 intervention subjects was $67,804 |
| Bajeux et al. 2022 | France | NA | 85 | From Acute Setting - Discharge Home, Nursing Home; Subacute care | 1. Medication Reconciliation- MR at admission (MRa) vs MR at discharge (MRd)- interview with the patient  2. DC planning- information handover/sharing with the patient at DC | - Not reported | - Multidisciplinary Team did not work together cohesively  - Difficulties in fully implementing complete MR process- 47.8% of intervention patients actually received the interview  highlighted difficulty in implementing long and complex med reconciliation processes | No significant difference in proportion of death, unplanned rehospitalisation and/or emergency visit for ADEs  Intervention patients more likely to feel that their discharge was well organised with better communication with community pharmacist No difference in patient’s medication knowledge |
| Dedhia et al. 2009 | United States | NA | 77 | From Acute Setting - Discharge Home | 1. Medication reconciliation- (no patient involvement)  2. DC planning- providing counselling on admission summary, medication recommendations and DC instructions through written information. Printed with larger font and used only simple language.  3. Contact information for hospitalist providers | - Multidisciplinary Team  - Intervention effectively incorporated into workflow | - Not sustainable in low/poor economic climate)  - Lengthy time spent with patient (1 hour per patient) - Not randomized | Decreased emergency department visits and readmission within 7 days (3% vs 10%) and (14% vs 21%) 30 days post DC |
| Huckfeldt et al. 2019 | United States | Patients high risk of re-admission | Did not report | From Acute Setting - Discharge Home | 1. Care plan (no patient involvement)  2. DC planning- conducted by a hospital home health nurse where education was provided and recommendations  3. Follow up- Home visit 96 hours post DC and weekly home or phone calls for 30 days post DC. Evaluate adherence | - Use of advance practice clinician | - Lack of resources - Did not incorporate GP in follow up | No statistically significant differences in readmission rates at 7 days and 30 days |
| Lazaro Cebas et al. 2022 | Spain | NA | 84 (I) 86 (C) | From Acute Setting - Discharge Home | 1. Medication Reconciliation- interview with patient at admission (HAMC list) 2. DC planning- Education to discuss treatment plan and written information given 3. Follow up- phone call 7- and 21-days post DC  to assess patient knowledge questionnaire | - Not reported | - Not reported | No statistical significance despite a decrease in readmission rates |
| Pellegrin et al. 2017 | United States | NA | Did not report | From Acute Setting - Discharge Home | 1. Medication review- identify and resolve any medication problems with patient during admission 2. DC planning- Counselling and education to explain changes made during admission 3. Follow up- to ensure understanding and promote adherence | - Collaboration between hospital and community pharmacist | - High workload  - Time limitations to complete intervention impede depth of education or counselling session | Statistically significant decrease in medication-related hospitalisations  Estimated annualized cost of avoided admissions was $6.6 million. |
| Steeman et al. 2006 | Belgium | Patients at risk of re-admission or institutionalization | 82 | From Acute Setting - Discharge Home; Subacute care | 1. DC planning- conducted by trained social worker or nurse using 'case management' method that aimed to provide continuity of care and promotion of self-care Care plan assessed done with patient 72 hours post admission and pre-DC | - Specialist healthcare professional involvement | - No follow-up  - Low positive predictive value of screening tool - Lack of specialist discharge managers | No statistical significance in readmission rates 15 and 90-days post discharge |
| White et al. 2013 | United States | Heart failure patients | 80 | From Acute Setting - Discharge Home; Nursing Home, subacute setting | 1. Education- provide by heart failure nurse through 1:1 session ranging from 15-120 minutes, handouts given (available in Multilanguage). Patients asked to 'teach-back' information and answer 4 questions 2. Follow up- phone call 7 days post DC to check medication knowledge (4 questions asked).  Re-education was provided if required | - Specialist healthcare professional involvement | - High workload – need adequate staffing to allow for patient teaching  - Lack of control group | No significant difference in 30-day hospital all-cause readmission rates among the patients answering correctly while hospitalized or during follow-up Greater time spent teaching was significantly associated with correctly answered questions |
| Mixed Methods | | | | | | | | |
| Kempen et al. 2019  MedBridge | Sweden | Intervention cohort participants of the MedBridge study | 78 | From Acute Setting - Discharge Home; Nursing Home; Subacute care | Semi-structured interviews centred around communication with the clinical pharmacist during hospital stay, decision‐making process within the CMR, information provided during hospital admission and follow-up practices. Interview conducted via phone 1 to 4 weeks post | - Not reported | - Not reported | Patients' experiences and views were positive overall  Patients expressed having an unclear understanding of the role of the ward-based pharmacist and problems with receiving and retaining information, may negatively impact the effectiveness of these interventions.  Expressed a limited role in decision‐making  Preferences for involvement in decision‐making varied substantially |
| Qualitative | | | | | | | | |
| Thevelin et al. 2022  OPERAM | Switzerland, Netherlands, Belgium, and Republic of Ireland | Intervention cohort participants of the OPERAM trial | 76 | From Acute Setting - Discharge Home; - Nursing Home; Subacute care | Semi-structured interviews and the Beliefs about Medicines Questionnaire (BMQ) embedded  Interviews conducted within 1-month post DC Interviews lasted on average 36min (range: 19–80 min) | - Not reported | - Limited participation - Clinicians neglecting patient needs and focusing solely on treating a disease - Too ill or too fatigued while hospitalised - Multiple clinician involved in care -  - Conflicting advice from different healthcare professionals | Patients generally displayed positive attitudes towards medication review, Emphasised importance of long-term, trusting relationships such as with their GPs for medication review.  Patients reported a lack of information and communication about medication changes and experienced paternalistic decision-making. |

**DC=discharge, SDM= shared decision ma*
